# Performance of Antigen Detection Tests for SARS-CoV-2: A Systematic Review and Meta-Analysis

**DOI:** 10.3390/diagnostics12061388

**Published:** 2022-06-04

**Authors:** Anastasia Tapari, Georgia G. Braliou, Maria Papaefthimiou, Helen Mavriki, Panagiota I. Kontou, Georgios K. Nikolopoulos, Pantelis G. Bagos

**Affiliations:** 1 Department of Computer Science and Biomedical Informatics, University of Thessaly, 35131 Lamia, Greece; atapari@dib.uth.gr (A.T.); gbraliou@dib.uth.gr (G.G.B.); mapapaef@gmail.com (M.P.); elenimavriki98@gmail.com (H.M.); pkontou@dib.uth.gr (P.I.K.); 2 Medical School, University of Cyprus, Nicosia 1678, Cyprus; gknikolopoulos@gmail.com

**Keywords:** COVID-19, SARS-CoV-2, antigen test, meta-analysis, diagnostic performance, sensitivity, specificity

## Abstract

Coronavirus disease 2019 (COVID-19) initiated global health care challenges such as the necessity for new diagnostic tests. Diagnosis by real-time PCR remains the gold-standard method, yet economical and technical issues prohibit its use in points of care (POC) or for repetitive tests in populations. A lot of effort has been exerted in developing, using, and validating antigen-based tests (ATs). Since individual studies focus on few methodological aspects of ATs, a comparison of different tests is needed. Herein, we perform a systematic review and meta-analysis of data from articles in PubMed, medRxiv and bioRxiv. The bivariate method for meta-analysis of diagnostic tests pooling sensitivities and specificities was used. Most of the AT types for SARS-CoV-2 were lateral flow immunoassays (LFIA), fluorescence immunoassays (FIA), and chemiluminescence enzyme immunoassays (CLEIA). We identified 235 articles containing data from 220,049 individuals. All ATs using nasopharyngeal samples show better performance than those with throat saliva (72% compared to 40%). Moreover, the rapid methods LFIA and FIA show about 10% lower sensitivity compared to the laboratory-based CLEIA method (72% compared to 82%). In addition, rapid ATs show higher sensitivity in symptomatic patients compared to asymptomatic patients, suggesting that viral load is a crucial parameter for ATs performed in POCs. Finally, all methods perform with very high specificity, reaching around 99%. LFIA tests, though with moderate sensitivity, appear as the most attractive method for use in POCs and for performing seroprevalence studies.

## 1. Introduction

COVID-19, caused by SARS-CoV-2, remains a global public health threat that has already claimed more than six million lives (https://covid19.who.int, accessed on 15 May 2022), with modeling estimates suggesting that this figure is probably much higher [1,2]. Vaccines, however, seem to perform well, especially after the administration of booster doses, providing moderate but short-lived protection from SARS-CoV-2 infection but significantly reducing COVID-19-related morbidity and mortality [3,4,5,6,7,8,9]. Non-pharmaceutical interventions (test-trace-isolate, hand washing, physical distancing, travel restrictions, school closures, closures of businesses, and stay-at-home orders) have also proved their effectiveness in containing the spread of the pandemic virus before the advent of vaccines [10,11,12]. Some of these measures will still be needed in our gradual efforts to return to normalcy. Testing in particular is essential to diagnosis, but also to developing and sustaining a reliable surveillance system for the years to come [13,14].

Real-time reverse transcription polymerase-chain-reaction (rt-PCR) test is the benchmark method for the clinical diagnosis of COVID-19 [15,16,17]. As such, it is designed for use with symptomatic people and has high analytical sensitivity. However, rt-PCR can detect viral genetic material even when the virus does not grow in a cell culture, suggesting that the presence of viral nucleic acid may not always reflect contagiousness. Moreover, it requires advanced laboratory equipment, specialist human resources, and significant infrastructure, often in a centralized setting, which increase costs, though these are less relevant for a single patient who needs a definite answer when he/she is tested. In summary, molecular diagnostic testing (nucleic acid amplification tests) becomes a less appealing method for frequent population screening to detect asymptomatic people with SARS-CoV-2 infection and as a tool to rapidly identify, contact-trace, and isolate highly infectious individuals. Antigen detection tests (AT) are immunoassays performed on pharyngeal, nasopharyngeal, nasal or throat swab specimens that detect the presence of a specific viral protein, which indicates viral activity [18,19]. The currently authorized AT include laboratory-based but also point-of-care (POC tests) and self-tests. AT are less expensive than rt-PCR, and most of them give results in approximately 15–30 min. In terms of weaknesses, AT are generally less sensitive than nucleic acid amplification tests. There are three main categories of AT used for the detection of SARS-CoV-2 infection. Lateral flow immunoassays (LFIA) are small, chromatography-based platforms used in POC. The sample is placed on the slot of the test plastic vector and an optical result (color) is obtained within 5–15 min [20]. Fluorescent immunoassays (FIA) are also small, handy, immunochromatography-based tests. The result is read by a fluorescence immunoassay analyzer within 5–20 min and can be performed in POC [21]. The chemiluminescence enzyme immunoassay (CLEIA) is a quick (about 30 min) and sensitive method to detect SARS-CoV-2 antigens. When the sample antigen reacts with the chemiluminescence substrate (antibody), the reaction product emits a photon of light instead of color development, which is read by an automated chemiluminescence analyzer [20].

Healthcare professionals, laboratory staff, and public health experts should comprehend the performance characteristics of AT, identify determinants of the accuracy of AT, and understand the differences among the three approaches to COVID-19-related testing (diagnostic, screening, and surveillance testing). In this respect, the aim of this meta-analysis is to comprehensively search the literature, to identify all relevant studies, to synthesize individual study estimates, and to determine the overall sensitivity and specificity of antigen-based methods for the detection of SARS-CoV-2, in comparison to quantitative rt-PCR (qPCR), for different types of clinical samples, and among both asymptomatic and symptomatic individuals.

## 2. Material and Methods

### 2.1. Literature Search Strategy

We conducted this systematic review and meta-analysis following the Preferred Reporting Items for Systematic Reviews and Meta-Analyses (PRISMA) guidelines [22] along with the advice for best practices [23]. We performed the literature search in Pubmed (https://pubmed.ncbi.nlm.nih.gov accessed on 15 May 2022), medRxiv (https://www.medrxiv.org accessed on 15 May 2022) and BioRxiv (https://www.biorxiv.org, accessed on 15 May 2022) up until 4 July 2021. The search terms were “(SARS-CoV-2 OR “Coronavirus disease 2019” OR COVID-19) AND antigen”. References from the selected studies were also scrutinized. Four independent researchers (AT, MP, HM, GB) evaluated search results; potential disagreements were resolved by discussion with GB and PB and consensus. Articles of all languages were considered to avoid gray literature publication bias [24].

### 2.2. Study Selection Criteria

Eligible criteria for inclusion in the meta-analysis were: (a) diagnosis of SARS-CoV-2 infection based on detection/quantitation of the viral genome by qPCR, according to World Health Organization (WHO)-, Centers for Disease Control (CDC)-, and European Centre for Disease Prevention and Control (ECDC)-approved methods [16,25,26,27]; (b) detection or measurement of nucleocapsid (N) or spike (S) proteins of SARS-CoV-2 (qualitatively or quantitatively depending on the method used); and (c) providing the necessary data that allow the calculation of sensitivity and specificity. We included studies that reported data on cases (positive samples) and healthy controls (negative samples) as well as studies with data available only for cases (see also Section 2.5).

### 2.3. Data Extraction

Data extraction was performed in a predetermined Microsoft Excel^®^ sheet. From each study we extracted the following information: first author’s last name, type of antigen used, type of sample, method of detection used, and the qPCR cycle threshold (Ct) values used for the detection of SARS-CoV-2 RNA. Additionally, the method of antigen testing used was recorded along with the brand name and the name of the manufacturer and the existence of data from the virus culture. Symptomatic and asymptomatic cases as well as male/female ratios were also recorded, if given. To obtain sensitivity and specificity measures, a 2 × 2 contingency table was constructed; thus, true positive (TP), false negative (FN), true negative (TN), and false positive (FP) results were recorded. In cases where no controls were used, we used TP and FN values only.

### 2.4. Study Outcomes

The primary outcome of this meta-analysis was the sensitivity and specificity of AT in relation to qPCR. Secondary outcomes included the performance of AT on different sample types (namely, nasopharyngeal, saliva, and throat samples) and by symptoms (asymptomatic versus symptomatic SARS-CoV-2 infected persons). We also explored the performance of AT across the number of qPCR Ct values (a higher Ct indicated lower viral load).

### 2.5. Data Analysis

The Quality Assessment of Diagnostic Accuracy Studies 2 (QUADAS-2 tool) was used to assess the quality of the included studies in terms of diagnostic accuracy [28]. The four domains assessed were patient selection, index test, reference standard, and flow and timing. Each domain was evaluated following classifications according to judgment, i.e., low risk, high risk, and unclear risk.

The bivariate meta-analytic method modified for the meta-analysis of diagnostic tests was used [29]. This method has been reported to be equivalent to the so-called hsROC method [30]. It uses logit-transforms of true positive rate (TPR) and false positive rate (FPR) in order to model sensitivity and specificity; it can also be used for the evaluation of between-studies variability (heterogeneity). Studies that include information only for logit (TPR)—that is, only for sensitivity—were included in the bivariate model under the missing at random (MAR) assumption in order to maximize statistical power and allow the modeling of between-studies variability and correlation [31]. Begg’s rank correlation test [32] and Egger’s regression test [33] were used on logit (TPR) to evaluate the presence of publication bias. Stata13 [34] was used to perform the analysis and run the command “mvmeta” with the method of moments for multivariate meta-analyses and meta-regression [35]. Statistical significance was set at *p* < 0.05; meta-analysis was performed when two or more studies were available, whereas tests for publication bias and meta-regression were performed when five or more studies were available.

## 3. Results

### 3.1. Characteristics of Studies

**Figure 1:**
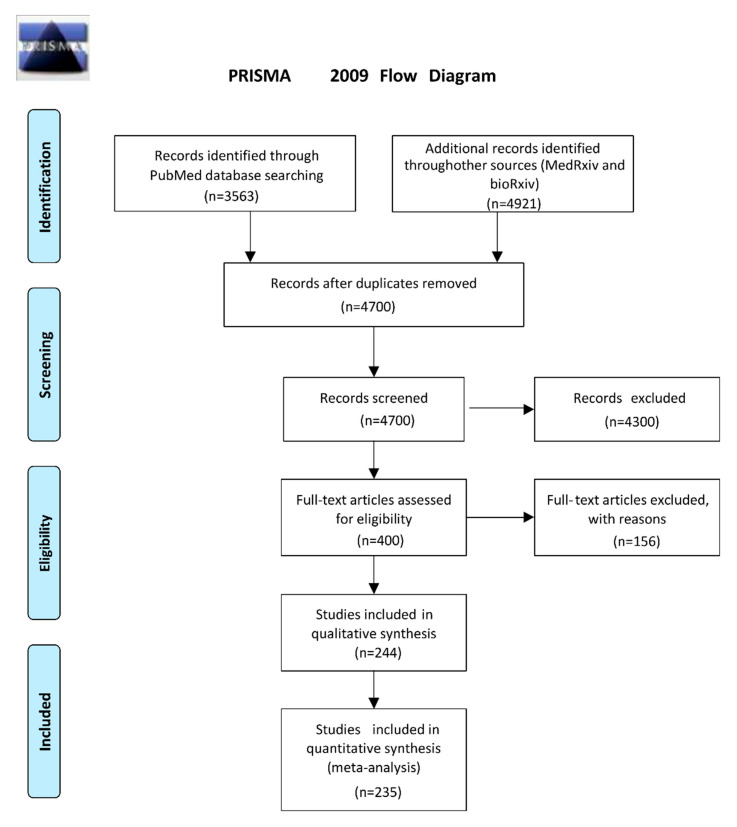
Preferred Reporting Items for Systematic Reviews and Meta-Analyses (PRISMA) flow diagram.

Following the literature search in Pubmed, MedRxiv, and BioRxiv by 4 July 2021, we retrieved 4700 unique articles (Figure 1). After scrutinizing abstracts and full papers and testing for eligibility criteria, we ended up with 235 articles, which included 31,387 SARS-CoV-2 infected individuals and 188,636 individuals without SARS-CoV-2 infection (total: 220,049 individuals). Two hundred and sixteen studies provided data on both cases and controls, while 19 studies reported results only for people with SARS-CoV-2 infection (Figure 1). Table 1 shows the characteristics of the included studies. All studies reported that SARS-CoV-2 infection was confirmed with qPCR of envelope (E), S or N protein according to WHO, CDC and ECDC guidelines. Various methods were used to identify or measure an antigen of SARS-CoV-2. The N antigen was investigated in 225 studies, the S antigen was investigated in eight studies, and in two studies, cumulative estimates were given for N + S or S + E + M (membrane) antigens. Four articles evaluated both N- and S-based assays. Most studies focused on rapid POC tests such as LFIA (181 studies), or FIA (38 studies). Chemiluminescence was used in 21 studies. In total, 83 different kits from 74 manufacturers and 18 in-house tests (LFIA, FIA, CLEIA) from the respective laboratories were used. Thirty-six studies used the same samples to compare different tests from different companies. Twelve studies used twelve unique techniques that are under development (LC-mass spectrometry [36,37], field-effect transistor (FET) based biosensing devices [38], organic electrochemical transistors-OECT [39], voltametric-based immunosensor [40], optical waveguide-based biosensor technology [41], deep learning-based surface-enhanced Raman spectroscopy [42], paper-based impedance sensor [43], high-field asymmetric waveform ion mobility spectrometry (FAIMS)–parallel reaction monitoring (PRM) [44], a colorimetric biosensor [45], an electrochemical glucose sensor [46], and a urine foaming test [47]). Finally, two studies were performed with urine samples [36,47]. Most studies used nasopharyngeal, nasal, pharyngeal, throat, oropharyngeal or saliva samples. We classified the samples into two groups, named “NSP”, containing the first three sample types, and “TS”, containing the last three types. The type of sample was clearly mentioned in 207 studies, while all types of samples were used without distinction in 31 studies. The results from different types of samples were compared with the same method in 11 studies. Finally, data from 60 studies on asymptomatic persons and 73 on symptomatic patients were also used to explore differences in diagnostic accuracy between these two patients’ groups. The results of the quality assessment of the research using the QUADAS tool are provided in Supplementary Table S1 and in Supplementary File S1.

### 3.2. Analysis of Diagnostic Performance

A great amount of the available data, for all methods, concerned samples detected with qPCR Ct values of 20, and mostly of 30 and 40. As shown in Table 2, the sensitivity of LFIA tests (using the N antigen) based on NSP samples that were qPCR-positive for Ct < 20 was 0.945 (95% CI: 0.930, 0.961). It declined, however, considerably to 0.329 (95% CI: 0.265, 0.393) for 30 < Ct < 40. LFIA tests using TS samples performed worse in terms of sensitivity, with a highest estimate of 0.805 (95% CI: 0.599, 1.000) in samples positive for Ct < 20 and a lowest of 0.085 (0.000, 0.176) for Ct > 30 (Table 2). The specificity of LFIA on NSP and TS samples (using the N antigen) was very high across all Ct intervals, ranging from 0.959 (95% CI: 0.923, 0.995) to 0.996 (95% CI: 0.993, 0.998). The sensitivity of FIA (using the N antigen) on NSP samples also showed a declining pattern from 0.935 (95% CI: 0.880, 0.990) for Ct < 20 to 0.435 (95% CI: 0.190, 0.680) for 30 < Ct < 40. Specificity was also very high using NSP qPCR positive samples for Ct < 30 (0.992, 95%: 0.979, 1.000). CLEIA (using the N antigen) had high sensitivity based on NSP samples that were PCR-positive for Ct < 30 (0.980, 95% CI: 0.960, 0.999); this estimate, however, was based on a smaller number of studies and dropped considerably at higher Ct (30–40) values (0.515; 95% CI: 0.220, 0.810). The specificity of CLEIA was very high in all comparisons. The evaluation of the performance of other methods (using the N antigen) on NSP and TS samples for the above studied Ct values intervals (0–20, 21–30, and 31–40) was based on a few studies but showed similar patterns. Data on methods using other antigens (i.e., based on S, E or M protein) were too scarce to allow reliable estimations (Table 2).

Combining all major methods (LFIA, FIA and CLEIA) on NSP and TS samples, measuring both N and S antigens and stratified according to two Ct values (<30 and <40), the maximum sensitivity was estimated at 0.858 (95% CI 0.835, 0.881) for NSP samples positive for Ct < 30 (Table 3). The sensitivity using qPCR positive NSP samples for Ct < 40 is lower at 0.726 (95% CI 0.706, 0.746). Again, antigen testing of NSP samples outperformed that of TS samples for both Ct < 30 and Ct < 40 (0.637 (95% CI: 0.478, 0.795) and 0.438 (95% CI: 0.332, 0.547), respectively). Specificity was very high in all meta-analyses (Table 3).

To attain a better insight into how each method performs, we compared the meta-analysis results for the sensitivity and specificity of each method (LFIA, FIA, CLEIA) on NSP and TS samples for all antigens cumulatively (N plus S). As shown in Table 3, in terms of sensitivity, the laboratory CLEIA method outperforms the point of care (POC) methods (LFIA and FIA), the NSP samples outperform the TS samples, and the best results are obtained for samples identified positive with PCR for Ct < 30 (0.977 (95% CI: 0.955, 0.998) versus 0.408 (95% CI: 0.292, 0.523) and 0.162 (95% CI: 0.083, 0.242)) (Table 3).

**Figure 2:**
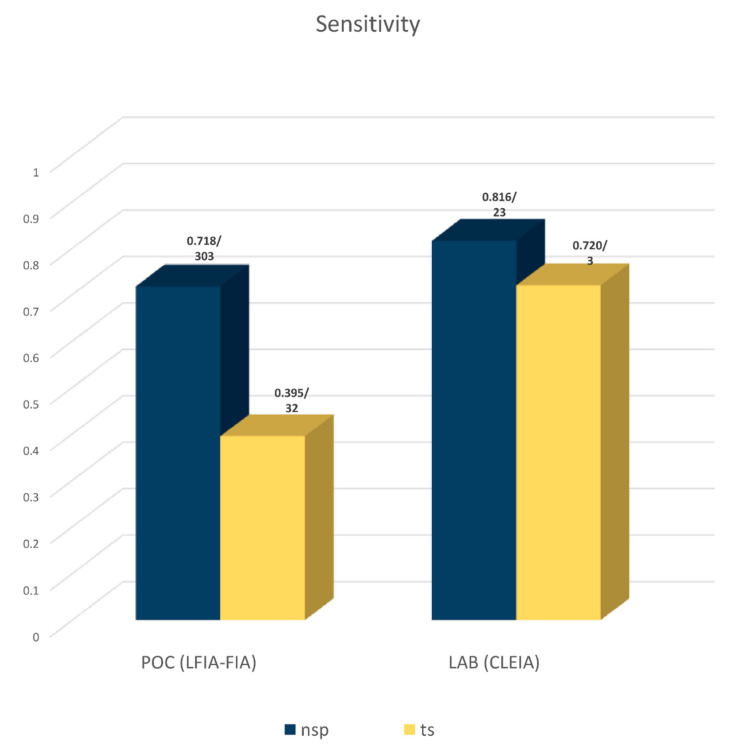
Performance of POC (LFIA and FIA) and laboratory (CLEIA) antigen-based methods in terms of sensitivity. All included assays in the meta-analysis use samples with Ct < 40 and test cumulatively both the nucleocapsid and Spike antigen. Numbers above the bars depict sensitivity values/number of studies included in each meta-analysis.

Since the ultimate goal of a diagnostic method for SARS-CoV-2 is to identify an infected person regardless of the low viral load, we compared the overall sensitivity of rapid tests performed in points either of care or where virus surveillance is performed (LFIA or FIA) with laboratory methods (CLEIA) that show the highest sensitivity. As shown in Figure 2 (and Table 3), the overall (for Ct < 40) sensitivity of POC methods is about 10% lower than that of the CLEIA method for NSP samples (0.718 (95% CI: 0.697, 0.739) compared to 0.816 (95% CI: 0.761, 0.870)). Specificity was again high in all cases ranging from 0.957 (95% CI: 0.889, 1.000) to 0.995 (95% CI: 0.993, 0.997), although due to the small number of the included studies in some subgroups, these results may have some uncertainty (Table 3).

To investigate the validity of our stratification analysis according to Ct values (<30 and <40), we tried to explore the association between a patient/sample’s infectivity and positivity in POC antigen tests (LFIA and FIA) and PCR tests using data from the included studies. We found 51 studies (Table 1) that used a virus culture to address this issue; however, the results were presented in a plethora of different ways and could not be quantitatively synthesized and analyzed, due to different reported parameters. From them, ten studies used virus cultures to only test the viral load (RNA copies/mL) that a POC test could detect. The remaining 34 studies presented a combination of data such as the limit of detection (LoD) in terms of RNA copies/mL or per swab or in pfus/mL, tissue culture infection dose (TCID), TCID50, TCID95%, sensitivity of POC tests in correlation with virus culture cytopathic effect (CPE) measured in different days and after zero, one or two passages. Nevertheless, sixteen studies [63,85,87,91,101,135,145,151,167,169,199,215,216,217,219,255] determined LoD Ct values ranging from 18.57 [219] to 34 [145], with most of them reporting Ct 30 as an average threshold for a POC test to be positive. Importantly, viral culture positivity (CPE), though measured under various protocols (directly [87,91,101,135,143,145,200,216,241] and indirectly [141,169,201,215,241,254]), has been extensively used as a marker for sample infectivity. Furthermore, twelve studies [54,76,85,143,170,199,213,217,233,235,237,241] presented data providing LoD values for a POC tests ranging from 5.10^3^ (Ct = 27.3 [63]) to 10^6^ RNA copies/swab (Ct = 30) [54,76]. Noteworthily, four studies on the CLEIA method [111,150,156,206] and four studies [41,44,46,47]) on in-house tests also investigated virus infectivity in correlation with either Ct values or positivity of these tests, but these were not analyzed since they were not reporting on POC tests. Taken together, the above observations suggest that if SARS-CoV-2-infected cell culture positivity is an indicator of a patient/sample that is likely to be infectious [202,258,259], this infectivity better correlates with POC test positivity than rt-PCR positivity. As we show herein, POC test positivity corresponds better to PCR positivity for Ct < 30; thus, POC tests are more likely to detect infectious individuals than positive PCR tests. 

**Figure 3:**
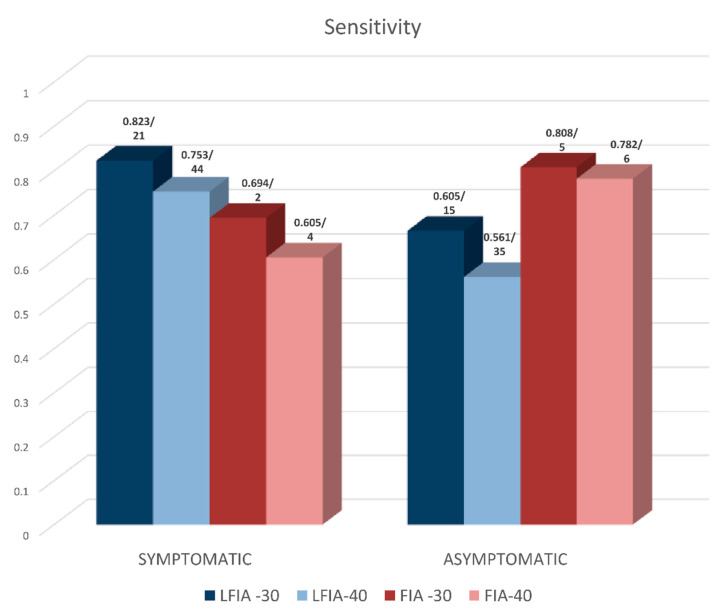
Performance of LFIA and FIA methods (N antigen-based) in terms of sensitivity on NSP samples in symptomatic vs. asymptomatic persons. Included assays in the meta-analysis are performed with positive samples for either Ct < 30 or Ct < 40. Numbers above the bars depict sensitivity values/number of studies included in each meta-analysis.

Additional meta-analysis showed that the sensitivity of LFIA (on NSP samples) in symptomatic patients was higher than that in asymptomatic individuals, both for Ct < 30 and Ct < 40 (symptomatic: 0.823 (95% CI: 0.765, 0.882) and 0.753 (95% CI: 0.713, 0.794)—asymptomatic: 0.665 (0.558, 0.772) and 0.561 (95% CI: 0.499, 0.622), respectively) (Table 4 and Figure 3). FIA assays seem to perform worse, but the meta-analysis estimates were based on a smaller number of studies. Specificity was very high for both LFIA and FIA methods (~99%) (Table 4).

## 4. Discussion

Test-trace-isolate remains a fundamental strategy to control SARS-CoV-2 transmission. Compared to PCR methods, antigen detection tests do not require specialized laboratory equipment and are less expensive, thus allowing repeated and point-of-care testing on a wide scale [18]. Our meta-analysis, summarizing evidence from thousands of people with and without SARS-CoV-2 infection diagnosed with rt-PCR, and performing various comparisons, shows that the overall performance of AT is comparable to rt-PCR, at least in terms of specificity, with meta-analytic estimates around 99%, irrespective of the method used. Sensitivity is lower and seems to depend on viral concentration being increased if detected at lower PCR cycles (Ct values). AT are also more sensitive when used on NSP samples and in symptomatic individuals. These updated findings are in accordance with previous efforts to summarize the evidence in this field [260,261]. Current best practices in meta-analysis suggest that a frequent update should be performed, and there is active research regarding the identification of the actual time that an update is needed [262,263]. As a matter of fact, previous works include statistical methods and surveillance systems that will identify the need for an update of a published meta-analysis [264,265]. More recently, the concept of a “living” systematic review has emerged, in which the review is continuously updated, incorporating relevant new data as they become available. Such reviews may be particularly important in fields where research evidence is emerging rapidly [266,267], and clearly, the COVID-19 pandemic is a perfect example of a field where new research accumulates in an unprecedented way and an updated meta-analysis is needed.

The sensitivity of AT is good but not ideal, and thus rt-PCR remains the gold standard for diagnosis. Given the suboptimal sensitivity of antigen tests, there is a likelihood of false negative results, which should be handled depending on the clinical and epidemiological circumstances. In general, confirmation of an AT result with rt-PCR in a laboratory is necessary when the result is not consistent with clinical and epidemiological information. Given their higher sensitivity among symptomatic people and in those with higher viral load (Ct < 30), ATs are expected to perform better when used for the diagnosis of SARS-CoV-2 infection in people with symptoms, in high-risk contacts of confirmed cases or in high-risk groups as health care workers with known exposure. Moreover, the sole detection of viral RNA with rt-PCR does not seem to overlap with patients’ infectiousness. Rather, POC (rapid) antigen tests that can only detect viral loads detectable with rt-PCR at Ct values <30 seem to more efficiently discriminate infectious SARS-CoV-2 carriers that should stay in isolation [202,255,258,259]. These findings are further supported by CDC recommendations, already posed by the end of 2020, which propose a Ct value of 33 as illustrative of contagiousness [204,268].

Proper interpretation of AT results is important not only for diagnosis but also for screening and surveillance purposes. This meta-analysis did not evaluate screening strategies that used AT. Nevertheless, it seems that AT can be used for regular screening of asymptomatic people in high-risk congregate settings, such as nursing homes, homeless shelters, detention facilities, etc., where the turnaround time of results is critical [269]. The fast identification of highly infected people in these facilities using rapid POC antigen tests will immediately inform infection prevention and control strategies and interventions, and consequently will significantly reduce onward transmission. Due to the lower sensitivity, screening in congregate high-risk settings but also mass screening may suffer from false negative results. Given the presumed direct correlation of rapid ATs’ positivity with patient’s infectivity, and the evidence that the effectiveness of screening depends more on frequency of testing and speed of reporting rather than on very high sensitivity [91,270], it seems that antigen tests can be used for repeated population screening.

In terms of specificity, AT performs extremely well, similarly to rt-PCR, thus minimizing the likelihood of false-positive results. However, false-positive results do occur, especially when the prevalence of SARS-CoV-2 infection in communities is low. This should be considered both in terms of diagnosis and when designing public health interventions or prevalence studies in low-prevalence settings because false positives result in a waste of resources (unnecessary isolation of cases and follow-up actions) and inaccurate estimations.

This meta-analysis is subject to the limitations of the individual studies. Bias and confounding at the study level cannot be easily addressed or corrected at the stage of meta-analysis. There are also issues that could affect the results and are usually not measured, reported, or addressed in studies that evaluate the accuracy of AT: storage and handling, reading of test results (time and interpretation), specimen collection and handling, time from specimen collection to testing, temperature of specimen, and potential cross-contamination, as was shown in the quality assessment of the research performed with the QUADAS tool.

We need to emphasize that the studies included in this meta-analysis were conducted before July 2021. Thus, data collection was completed at a time prior to the emergence of the Omicron variant and thus, the conclusions drawn from this work involve mainly the initial Wuhan strain, Alpha, Beta and Delta (to some extent) variants. A complete treatment of the question regarding the effectiveness of antigen tests against the newly emerged Omicron variant [271] would require a study of its own, but nevertheless we might be able to highlight some of the available evidence. Initially, there were concerns regarding the effectiveness of the tests [272], but the first report with the Abbott BinaxNow SARS-CoV-2 Rapid Antigen Assay provided evidence that it can be used efficiently [273]. Similar results were reported with another approved test (E25Bio, Inc., Cambridge, MA, USA, and Perkin Elmer, Waltham, MA, USA) in a comparison study of the Alpha, Gamma, Delta and Omicron variants [274], and for Panbio™ COVID-19 Ag Rapid Test [275]. Stanley and coworkers examined the analytical sensitivity of the Abbott BinaxNow, the AccessBio CareStart and LumiraDx antigen tests, and found that the level of detection was at least as good for Omicron as for the initial Wuhan strain [276]. Finally, Deerain and coworkers measured the sensitivity of ten different lateral flow devices against the omicron variant and found that the analytical sensitivities of these ten kits were similar for both the Delta and Omicron variants [277]. All in all, even though more studies are needed, the available evidence suggests that the currently used ATs can be used efficiently for detecting the Omicron variant and large discrepancies in sensitivity due to its spread are not expected.

Finally, evaluation of different testing strategies in various settings is also urgently needed [278]. Moreover, the lack of an agreed, universal, standardized protocol starting from specimen collection and handling to performing and reading the test and to the way(s) that its performance is validated (rt-PCR (genes, Ct values) or cytopathic effects of virus cultures (reference virus strain) or RNA copies, etc. [140,279]) has also been revealed through our current systematic review and meta-analysis. Only in such uniform settings can accurate comparisons of methods and individual tests be performed in order to optimally track and manage SARS-CoV-2 infection in the global community.

## Figures and Tables

**Table 1:** Characteristics of the 235 studies included in the meta-analysis.

Author	Country of Study	Ag	Type of Sample	Ag Detection Method/Virus Culture Data	Kit Name	Kit Company	Ct Values Tested	Signal Detection [Rapid (w/wo Detector)/Quick]	Total Individuals	Cases	Controls
Mak et al. [48]	China	N	1. nsp 2. ts	1. LFIA 2. LFIA 3. LFIA/virus culture data	1. COVID-19 Ag Respi-Strip 2. NADAL COVID-19 Ag Test 3. Standard Q COVID-19 Ag	1. Coris Bioconcept, Belgium 2. Nal Von Minden GmbH, Germany 3. SD Biosensor, Korea	up to 20/up to 30/up to 40/0–20/20–30/30–40	Rapid	35	35	NA
Linares et al. [49]	Spain	N	nsp	LFIA	Panbio COVID-19 Ag Rapid Test Device	Abbott Rapid Diagnostic Jena GmbH, Jena, Germany	Up to 40	Rapid	255	60	NA
Gupta et al. [50]	India	N	nsp	LFIA	Standard Q rapid antigen detection test	SD Biosensor, Inc., Gurugram	Up to 40	Rapid	330	77	253
Fenollar et al. [51]	France	N	nsp	LFIA	PANBIO COVID-19 Ag rapid test device	Abbott, USA	Up to 40	Rapid	341	204	137
Nalumansi et al. [52]	Uganda	N	nsp	LFIA	STANDARD Q COVID-19 Ag Test	SD -Biosensor, Republic of Korea	Up to 30/up to 40/30–40	Rapid	262	90	172
Parada-Ricart et al. [53]	Spain	N	nsp	FIA	2019-nCoV Antigen Rapid Test Kit (FIA)	Shenzhen Bioeasy Biotechnology CO LTD, China	Up to 40	Rapid/detector	172	26	146
Lee et al. [54]	Korea	S	nsp	LFIA/virus culture data	In-house test		Up to 40	Rapid/detector	8	3	5
Cerutti et al. [55]	Italy	N	nsp	LFIA	STANDARD Q COVID19 Ag	SD-Biosensor, RELAB, I	Up to 40	Rapid	330	109	221
Diao et al. [56]	China	N	nsp	FIA	In-house test		Up to 40	Rapid/detector	502	356	146
Young et al. [57]	USA	N	nsp	1. LFIA 2. FIA	1. BD Veritor™ System 2. Sofia 2 SARS Antigen FIA	1. Becton-Dickinson and Company, USA 2. Quidel, San Diego, CA	Up to 40	1. Rapid/optional detector 2. Rapid/detector	612	81	531
Liotti et al. [58]	Italy	N	nsp	FIA	STANDARD F COVID19 Ag (FIA)	SD Biosensor, Suwon, Korea	Up to 20/up to 30/up to 40/0–20	Rapid/detector	359	104	255
Ogawa et al. [59]	Japan	N	Nsp	CLEIA	Lumipulse SARS-CoV-2 Ag	Fujirebio, Tokyo, Japan	Up to 40	Detector	325	24	301
Hirotsu et al. [60]	Japan	N	nsp	CLEIA	LUMIPULSE SARS-CoV-2 Ag kit	Fujirebio, Inc. (Tokyo, Japan)	Up to 40	Detector	313	58	255
Nagura-Ikeda et al. [61]	Japan	N	ts	LFIA	Espline SARS-CoV-2	Fuji Rebio Inc.	Up to 40	Rapid	103	84	19
Mak et al. [62]	Hong Kong	N	1. nsp/ts 2. ts	LFIA	BIOCREDIT COVID-19 Ag kit	RapiGEN Inc.	Up to 20/up to 30/up to 40/0–20/20–30	Rapid optional detector	160	51	109
Mertens et al. [63]	Belgium	N	nsp	LFIA/virus culture data	COVID-19 Ag RespiStrip	Coris BioConcept	Up to 30/up to 40	Rapid	328	132	196
Blairon et al. [64]	Belgium	N	nsp	LFIA	COVID-19 Ag Respi-Strip	Coris Bioconcept, Gembloux, Belgium	Up to 40	Rapid	774	159	615
Porte et al. [21]	Chile	N	nsp/ts	FIA	2019-nCoV Antigen Rapid Test Kit (FIA)	Bioeasy Biotechnology Co., Shenzhen, China	Up to 30	Rapid/detector	127	82	45
Scohy et al. [65]	Belgium	N	nsp	LFIA	COVID-19 Ag Respi-Strip	Coris BioConcept, Gembloux, Belgium	Up to 40	Rapid	148	106	62
Lambert-Niclot et al. [66]	France	N	nsp	LFIA	COVID-19 Ag Respi-Strip	Coris BioConcept, Gembloux, Belgium	Up to 40	Rapid	138	94	44
Diao et al. [67]	China	N	nsp	FIA	In-house test		Up to 30/up to 40/30–40	Rapid	239	208	31
Beck et al. [68]	Milwaukee	N	nsp	FIA	Sofia SARS FIA test (SOFIA)	Quidel, San Diego, CA	Up to 40	Rapid/detector	346	61	285
Krüttgen et al. [69]	Germany	N	nsp	LFIA	SARS-CoV-2 Rapid Antigen Test	Roche, Switzerland	Up to 20/up to 30/up to 40/0–20/20–30/30–40	Rapid	150	75	75
Albert et al. [70]	Spain	N	nsp	LFIA/virus culture data	Panbio™ COVID-19 Ag Rapid Test Device	Abbott Diagnostic GmbH, Jena, Germany	Up to 40	Rapid	412	54	358
Chaimayo et al. [71]	Thailand	N	nsp/ts	LFIA	Standard Q COVID-19 Ag test	SD Biosensor^®^, Chuncheongbuk-do, Republic of Korea	Up to 40	Rapid	454	60	394
Lanser et al. [72]	Austria	N	nsp	LFIA	Panbio™ COVID-19 Ag Rapid test	Abbott, Chicago, Illinοis	Up to 30/up to 40/30–40	Rapid	53	51	2
Gremmels et al. [73]	The Netherlands/Aruba	N	nsp	LFIA	Panbio COVID-19 Ag rapid test device	Abbott, Lake Country, IL, USA	Up to 40	Rapid	2948	202	2746
Drevinek et al. [74]	Czech Republic	N	nsp	1. LFIA 2. FIA	1. Panbio COVID-19 Ag Rapid Test 2. Standard F COVID-19 Ag FIA	1. Abbott, Germany 2. SD Biosensor, Republic of Korea	Up to 20/up to 30/up to 40/0–20/20–30/30–40	1. Rapid 2. Rapid/detector	591	223	368
Schwob et al. [75]	Switzerland	N	nsp	1. LFIA 2. LFIA 3. LFIA	1. STANDARD Q COVID-19 Ag Test 2. Panbio COVID-19 Ag Test 3. COVID-VIRO	1. SD -Biosensor, Republik of Korea 2. Abbott, Germany 3. AAZ-LMB	Up to 40	Rapid	928	372	556
Corman et al. [76]	Germany	N	nsp	1. LFIA 2. LFIA 3. LFIA 4. LFIA 5. LFIA 6. LFIA 7. LFIA/virus culture data	1. Panbio COVID-19 Ag Test 2. BIOCREDIT COVID-19 Ag kit 3. Coronavirus Ag Rapid Test Cassette (swab) 4. COVID-19 Ag Respi-Strip 5. RIDA^®^QUICK SARS-CoV-2 antigen 6. NADAL COVID19-Ag Test 7. SARS-CoV-2 Rapid Antigen Test	1. Abbott, Germany 2. RapiGEN Inc. 3. Healgen 4. Coris Bioconcept, Gembloux, Belgium 5. R-Biopharm 6. NAL von minden 7. Roche	Up to 40	Rapid	150	115	35
Abdulrahman et al. [77]	Bahrain	N	nsp	LFIA	Panbio COVID 19 antigen rapid test device	Abbott Rapid Diagnostic Jena GmbH, Jena, Germany	Up to 30	Rapid	4183	733	3450
Yokota et al. [78]	Japan	N	Nsp, ts	1. LFIA 2. CLEIA	1. Espline SARS-CoV-2 2. Lumipulse SARS-CoV-2 Ag kit	1. Fujirebio, Tokyo, Japan 2. Fujirebio, Tokyo, Japan	Up to 30/up to 40/20–30	1. Rapid 2. Quick/detector	34	34	NA
Nash et al. [79]	USA/Brazil	1. N 2. S	nsp	LFIA	In-house		Up to 20/up to 30/up to 40/0–20/20–30/30–40	Rapid	311	172	139
Van der Moeren et al. [80]	The Netherlands	N	nsp	LFIA	BD Veritor™ System	Becton-Dickinson and Company, USA	Up to 20/up to 30/up to 40/0–20/20–30	Rapid/optional detector	351	17	334
Porte et al. [81]	Chile	N	nsp/ts	1. FIA 2. FIA	1. SOFIA SARS Antigen FIA 2. STANDARD^®^ F COVID-19 Ag FIA	1. Quidel Corporation, San Diego, CA, USA 2. SD Biosensor Inc., Gyeonggi-do, Republic of Korea	Up to 30/up to 40/30–40	Rapid/detector	91	59	32
Krüger et al. [82]	Germany/UK	N	nsp/ts	1. FIA 2. LFIA 3. LFIA/virus culture data	1. 2019-nCoV Ag Fluorescence Rapid Test Kit 2. COVID-19 Ag Respi-Strip 3. STANDARD Q COVID-19 Ag Test	1. Shenzhen Bioeasy Biotechnology Co. Ltd., Guangdong Province, China 2. Coris Bioconcept, Gembloux, Belgium 3. SD Biosensor, Inc., Gyeonggi-do, Korea	Up to 30/up to 40/30–40	1. Rapid/detector 2. Rapid 3. Rapid	2407	72	2335
Singh et al. [46]	San Diego	S	nsp	ECGluS	In-house		Up to 40	Quick *	24	16	8
Ventura et al. [83]	Italy	S + E + M	Nsp/ts	CBS	In-house		Up to 40	Detector	94	45	49
Herrera et al. [84]	Florida	N	nsp	LFIA	NR/AdventHealth Centra Care		Up to 40	Rapid	1669	486	1183
Renuse et al. [44]	USA	N	nsp	FAIMS-PRM	In-house		Up to 40	Detector	176	88	88
Pickering et al. [85]	UK	N	nsp-ts	LFIA/virus culture data	1. Innova Rapid SARS-CoV-2 Antigen Test 2. Spring Healthcare SARS-CoV-2 Antigen Rapid Test Cassette 3. E25Bio Rapid Diagnostic Test 4. Encode SARS-CoV-2 Antigen Rapid Test Device 5. SureScreen COVID-19 Rapid Antigen Test Cassette	1. Xiamen Biotime Biotechnology, Fujian, China 2. Shanghai ZJ Bio-Tech, Shanghai, China 3. E25Bio, Cambridge, MA, USA 4. Zhuhai Encode Medical Engineering, Zhuhai, China 5. SureScreen Diagnostics, Derby, UK	20–30	Rapid	200	100	100
Harmon et al. [86]	Washington	N	nsp	FIA	Sofia-2 SARS-CoV-2 Antigen Tests	Quidel, San Diego, CA	Up to 40	Rapid/detector	23,462	83	23,379
Korenkov et al. [87]	Germany	N	nsp-ts	LFIA/virus culture data	STANDARD Q COVID-19 Ag Test	SD Biosensor, Inc., Gyeonggi-do, Korea	Up to 20/up to 30/up to 40/0–20/20–30/30–40	Rapid	2028	210	1818
Ehsan et al. [43]	Saudi Arabia	S	nsp	Paper-based impedance sensor	In-house		Up to 40	Detector	5	3	2
Seynaeve et al. [88]	Belgium	N	nsp	LFIA	1. COVID-19 Ag Respi-Strip 2. coronavirus antigen rapid test cassette	1. Coris Bioconcept, Belgium 2. Healgen Scientific, LLC, USA	Up to 30/ Up to 40/30–40	Rapid	163	98	65
Di Domenico et al. [89]	Italy	1. N 2. S	1. nsp 2. ts	1. ELISA based 2. LFIA/virus culture data	1. Portable COVID-19 Antigen Lab Test 2. Panbio™ COVID-19 Ag Rapid Test Device	1. Stark 2. Abbott Diagnostic GmbH, Jena, Germany	Up to 40	Rapid	433	36	397
Kiro et al. [90]	India	N	nsp	FIA	STANDARD^®^ F COVID-19 Ag FIA	SD Biosensor Inc., Gyeonggi-do, Republic of Korea	Up to 40	Rapid/detector	354	136	218
Smith et al. [91]	Illinois	N	1. nsp-ts 2. nsp	FIA/virus culture data	SOFIA SARS Antigen FIA	Quidel Corporation, San Diego, CA, USA	Up to 40	Rapid/detector	43	43	NA
L’Huillier et al. [92]	Switzerland	N	nsp	LFIA	Panbio™ COVID-19 Ag Rapid Test Device	Abbott Diagnostic GmbH, Jena, German	Up to 40	Rapid	822	119	703
Gupta et al. [93]	India	S	nsp	ELISA	In-house		Up to 40	Quick	232	44	188
Wagenhäuser et al. [94]	Germany	N	ts	LFIA	1. NADAL COVID-19 Ag Test 2. Panbio COVID-19 Ag rapid test device 3. MEDsan SARS-CoV-2 Antigen Rapid Test	1. Nal Von Minden GmbH, Germany 2. Abbott Laboratories, Abbott Park IL, USA 3. MEDsan GmbH, Hamburg, Germany	Up to 40	Rapid	5056	101	4955
Fernandez et al. [95]	Spain	N	nsp	FIA	LumiraDx™	LumiraDx™ Limited, Londres, Reino Unido	Up to 40	Rapid/detector	46	24	22
Amer et al. [96]	Egypt	N	nsp-ts	LFIA	STANDARD Q COVID-19 Ag Test	SD Biosensor, Inc., Gyeonggi-do, Korea	Up to 40	Rapid	47	45	2
Baccani et al. [97]	Italy	N	nsp	1. CLEIA 2. FIA 3. FIA	1. Lumipulse G SARS-CoV-2 Ag 2. STANDARD^®^ F COVID-19 Ag FIA 3. AFIAS COVID-19 Ag	1. Fujirebio, Tokio, Japan 2. SD Biosensor; Suwon-si, Korea 3. Menarini; Florence, Italy	Up to 30/Up to 40/30–40	1. Quick/detector 2. Rapid/detector 3. Rapid/detector	375	85	290
Matsuzaki et al. [98]	Japan	N	nsp	CLEIA	1. VITROS^®^ SARS-CoV-2 Antigen Test 2. LUMIPULSE^®^ SASR-CoV-2 Ag Test	2. Ortho Clinical Diagnostics, Rochester, NY, USA 3. Fujirebio, Tokio, Japan	Up to 40	1. Quick/detector 2. Quick/ detector	128	49	79
Jakobsen et al. [99]	Denmark	N	nsp	LFIA	STANDARD Q COVID-19 Ag Test	SD Biosensor, Inc., Gyeonggi-do, Korea	Up to 40	Rapid	4811	221	4590
Ngo Nsoga et al. [100]	Switzerland	N	nsp-ts	LFIA/virus culture data	Panbio™ COVID-19 Ag Rapid Test Device	Abbott Diagnostic GmbH, Jena, German	Up to 40	Rapid	402	168	234
Funabashi et al. [41]	Japan	N	nsp	Optical waveguide-based biosensor technology	In-house		Up to 40	Detector	64	34	30
Smith et al. [101]	Maryland	N	nsp	FIA	SOFIA SARS Antigen FIA	Quidel Corporation, San Diego, CA, USA	Up to 40	Rapid/detector	2887	235	2652
Eleftheriou et al. [102]	Greece	N	nsp	LFIA	Panbio™ COVID-19 Ag Rapid Test Device	Abbott Diagnostic GmbH, Jena, German	Up to 40	Rapid	744	51	693
Huang et al. [42]	China	S	ts	Deep learning-based surface-enhanced Raman spectroscopy	In-house		Up to 40	NA/detector	57	30	27
Lindner et al. [103]	Germany	N	nsp-ts	LFIA	STANDARD Q COVID-19 Ag Test	SD Biosensor, Inc., Gyeonggi-do, Korea	Up to 20/Up to 30/Up to 40/0–20/20–30/30–40	Rapid	146	40	106
Ferte et al. [104]	France	N	nsp	LFIA	Panbio™ COVID-19 Ag Rapid Test Device	Abbott Diagnostic GmbH, Jena, German	Up to 40	Rapid	688	52	636
Fernandez-Montero et al. [105]	Spain	N	nsp-ts	LFIA	SARS-CoV-2 Rapid Antigen Test	Roche	Up to 20/Up to 30/Up to 40/0–20/20–30/30–40	Rapid	2543	49	2494
Hoehl et al. [106]	Germany	N	nsp	LFIA	RIDA^®^QUICK SARS-CoV-2 Antigen	R-Biopharm AG	Up to 30	Rapid	9	9	NA
Lee et al. [107]	Korea	N	nsp	LFIA	STANDARD Q COVID-19 Ag Test	SD Biosensor, Inc., Gyeonggi-do, Korea	Up to 20/Up to 30/Up to 40/0–20/20–30/30–40	Rapid	680	380	300
Mayanskiy et al. [108]	Russia	N	nsp	ELISA	CoviNAg EIA	XEMA, Russia	Up to 20/Up to 30/Up to 40/0–20/20–30/30–40	Detector	277	182	95
Leixner et al. [109]	Austria	N	nsp	LFIA	AMP Rapid Test SARS-CoV-2 Ag	AMP Diagnostics, AMEDA Labordiagnostik GmbH, Graz, Austria	Up to 30/Up to 40/30–40	Rapid	392	94	298
Hirotsu et al. [110]	Japan	N	nsp	1. CLEIA 2. CLEIA	1. LUMIPULSE^®^ SASR-CoV-2 Ag Test 2. Elecsys1 SARS-CoV-2 Antigen Assay	1. Fujirebio, Tokio, Japan 2. Roche, Basel, Switzerland	Up to 40	Detector	637	487	150
Chavan et al. [36]	USA	N	urine	mass spectrometry	In-house		Up to 40	Detector	50	39	11
Fiedler et al. [111]	Germany	N	nsp	CLEIA/virus culture data	LIAISON^®^ SARS-CoV-2 Ag	DiaSorin	Up to 40	Detector	182	110	72
Dierks et al. [112]	Germany	N	nsp	1. FIA 2. LFIA	1. LumiraDx™ 2. NADAL COVID-19 Ag Test	1. LumiraDx™ Limited, London, United Kingdom 2. Nal Von Minden GmbH, Germany	Up to 40	1. Rapid/detector 2. Rapid	444	11	433
Terpos et al. [113]	Slovenia	N	nsp	LFIA	COVID-19 Antigen Detection Kit (Colloidal Gold)	Zhuhai Lituo Biotechnology Co., Ltd.	Up to 30/Up to 40/30–40	Rapid	358	114	244
Osmanodja et al. [114]	Germany	N	nsp-ts	LFIA	Dräger Antigen Test SARS-CoV-2	Dräger Safety AG and Co. KGaA, Lübeck, Germany	Up to 20/Up to 30/Up to 40/0–20/20–30/30–40	Rapid	379	70	309
Harris et al. [115]	USA	N	nsp	FIA	SOFIA SARS Antigen FIA	Quidel Corporation, San Diego, CA, USA	Up to 30/Up to 40/30–40	Rapid/detector	2429	324	2105
Cento et al. [116]	Italy	N	nsp	FIA	LumiraDx™	LumiraDx™ Limited, Londres, Reino Unido	Up to 30/Up to 40/30–40	Rapid/detector	960	347	613
Kumar et al. [117]	India	N	nsp	LFIA	STANDARD Q COVID-19 Ag Test	SD Biosensor, Inc., Gyeonggi-do, Korea	Up to 40	Rapid	6	6	NA
Orsi et al. [118]	Italy	N	nsp	FIA	1. FREND™ COVID-19 Ag 2. STANDARD^®^ F COVID-19 Ag FIA	1. NanoEntek, Korea 2. SD Biosensor; Suwon-si, Korea	Up to 30/Up to 40/30–40	Rapid/detector	110	60	50
Blairon et al. [119]	Belgium	N	nsp	LFIA/virus culture data	1. Coronavirus Ag Rapid Test Cassette 2. GSD NovaGen SARS-CoV-2 (COVID-19) Antigen Rapid Test 3. Aegle Coronavirus Ag Rapid Test Cassette	1. BioRad 2. NovaTec Immunodiagnostica GmbH 3. LumiraDx	Up to 20/Up to 30/Up to 40/0–20/20–30/30–40	Rapid	199	97	102
Bornemann et al. [120]	Germany	N	nsp	FIA	SOFIA SARS Antigen FIA	Quidel Corporation, San Diego, CA, USA	Up to 30/Up to 40/30–40	Rapid/detector	1391	91	1300
Kruger et al. [121]	Germany	N	1. nsp 2. ts 3. nsp-ts	LFIA	Panbio™ COVID-19 Ag Rapid Test Device	Abbott Diagnostic GmbH, Jena, German	Up to 30/Up to 40/30–40	Rapid	1108	106	1002
Eissa et al. [40]	Saudi Arabia	N	nsp	Voltammetric-based immunosensor	In-house		Up to 30/Up to 40/30–40	Detector	6	5	1
Shaikh et al. [122]	USA	N	nsp	LFIA	BinaxNOWTM COVID-19 Ag Card	Abbott Diagnostics Scarborough, Inc., USA	Up to 40	Rapid	199	39	160
Diez Flecha et al. [123]	Spain	N	nsp	LFIA	Panbio™ COVID-19 Ag Rapid Test Device	Abbott Diagnostic GmbH, Jena, German	Up to 30/Up to 40/30–40	Rapid	55	49	6
Yokota et al. [124]	Japan	N	ts	CLEIA	In-house		Up to 40	detector	2056	89	1967
Guo et al. [39]	Saudi Arabia	N	1. nsp 2. ts 3. nsp-ts	OECT	In-house		Up to 40	detector	24	11	13
Klein et al. [125]	Germany	N	nsp	LFIA	Panbio™ Ag-RDT	Abbott Diagnostics, Jena, Germany	Up to 20/Up to 30/Up to 40/0–20/20–30/30–40	Rapid	290	39	251
Caramello et al. [126]	Italy	N	nsp	1. LFIA 2. FIA	1. SD BIOSENSOR Ag-RDT 2. LUMIRADX Ag-RDT	1. SD BIOSENSOR Ag-RDT 2. LumiraDx UK Ltd., Dumyat Business Park, Alloa, FK10 2PB, UK)	Up to 40	1. Rapid 2. Rapid/detector	324	210	114
Koeleman et al. [127]	Netherlands	N	nsp-ts	LFIA	1. Certest SARS-CoV-2 2. Roche SARS-CoV-2 Rapid Antigen Test 3. Romed Coronavirus Ag Rapid Test 4. BD Veritor SARS-CoV-2 point-of-care test 5. Panbio™ COVID-19 Antigen rapid test	1. Certest Biotec S.L., Spain 2. Roche, Switzerland 3. Romed, The Netherlands 4. Becton, Dickinson and Company, USA 5. Abbott, USA	Up to 40	Rapid	980	340	640
Šterbenc et al. [128]	Slovenia	N	nsp	LFIA	SARS-CoV-2 rapid antigen test (Roche)	Roche Diagnostics GmbH, Mannheim, Germany)	Up to 40	Rapid	191	2	189
Kumar et al. [129]	India	N	nsp-ts	FIA	STANDARD™ Q COVID-19 Ag test kit	SD Biosensor; Suwon-si, Korea	Up to 40	Rapid/detector	204	12	192
Soleimani et al. [130]	Belgium	N	nsp	FIA	1. COVID19Speed-antigen test 2. Panbio™ COVID-19 Ag rapid test	1. BioSpeedia 2. Abbott	Up to 30/Up to 40/30–40	Rapid/detector	401	196	205
Takeuchi et al. [131]	Japan	N	nsp	LFIA	QuickNavi-COVID19 Ag	Denka Co., Ltd., Tokyo, Japan	Up to 30	Rapid	862	51	811
Linares et al. [49]	Spain	N	nsp	1. LFIA 2. FIA	1. Panbio COVID-19 Ag Rapid Test Device 2. D-Biosensor STANDARD F COVID-19 Ag	1. Abbot Rapid Diagnostics GmbH, Jena 2. SD Biosensor, Inc.	Up to 20/Up to 30/20–30	1. Rapid 2. Rapid/detector	356	170	186
Homza et al. [132]	Czech Republic	N	nsp	LFIA	Ecotest COVID-19 Antigen Rapid Test	Assure Tech, Hangzhou, China	Up to 20/Up to 30/Up to 40/0–20/20–30/30–40	Rapid	491	164	327
Van der Moeren et al. [133]	Netherlands	N	nsp-ts	CLEIA	BD veritor system for rapid detection of SARS-CoV-2 (VRD)	Becton-Dickinson and Company, USA	20–30	Detector	978	161	817
Brihn et al. [134]	USA	N	nsp	FIA	Quidel Sofia 2 SARS Antigen Fluorescent Immunoassay	Quidel Corporation	Up to 30	Rapid/detector	2039	149	1890
Nordgren et al. [135]	Sweden	N	nsp	LFIA/virus culture data	1. Panbio™ COVID-19 Ag Rapid Test 2. Zhejiang Orient Gene	1. Abbott 2. Healgen Biotech Coronavirus Ag rapid test cassette	Up to 20/Up to 40/20–30	Rapid	462	156	306
Holzner et al. [136]	Germany	N	nsp	LFIA	Standard Q COVID-19 Ag	SD Biosensor, Korea	Up to 30	Rapid	2280	456	1824
Kim et al. [137]	Korea	N	nsp	LFIA	GenBody COVID-19 Ag Test (COVAG025)	GenBody Inc.	Up to 40/20–30	Rapid	330	130	200
Bianco et al. [138]	Italy	N	nsp	FIA	LumiraDx™ SARS-CoV-2 Antigen Test	LumiraDx	30–40	Rapid/detector	907	298	609
Peña et al. [139]	Chile	N	nsp	LFIA	SARS-CoV-2 RAT	SD Biosensor	Up to 30	Rapid	842	73	769
Muhi et al. [140]	Australia	N	nsp	LFIA/virus culture data	PanBioTM COVID-19 Ag	Abbott	Up to 40	Rapid	189	26	163
Uwamino et al. [141]	Japan	N	nsp	LFIA/virus culture data	Espline SARS-CoV-2 RAD	FUJIREBIO, Tokyo, Japan	Up to 40	Rapid	117	25	92
Thakur et al. [142]	India	N	nsp-ts	LFIA	PathoCatch	ACCUCARE	20–30	Rapid	677	84	593
Homza et al. [143]	Czech Republic	N	nsp	LFIA/virus culture data	1. SARS-CoV-2 Antigen Rapid Test Kit 2. Ecotest COVID-19 Antigen Rapid Test 3. Standard Q COVID-19 Ag 4. Immupass VivaDiag™ SARS-CoV-2 Ag Rapid Test 5. ND COVID-19 Ag test	1. JOYSBIO (Tianjin) Biotechnology Co., Ltd., Tianjin, China 2. Assure Tech, Hangzhou, China 3. SD Biosensor, Korea 4. VivaChek Biotech (Hangzhou) Co., Ltd., Hangzhou, China 5. NDFOS, Eumseong, Korea	Up to 40	Rapid	1141	407	734
Shah et al. [144]	USA	N	nsp	LFIA	BinaxNOW COVID-19 Ag	Abbott	20–30	Rapid	2110	334	1776
McKay [145]	USA	N	nsp	LFIA/virus culture data	BinaxNOW Rapid Antigen Test	Abbott	Up to 40	Rapid	532	105	427
Yin et al. [146]	Belgium	N	nsp	LFIA	1. Panbio™ COVID-19 Ag Rapid Test Device 2. BD Veritor™ SARS-CoV-2 3. COVID-19 Ag Respi-Strip 4. SARS-CoV-2 Rapid Antigen Test	1. Abbott Rapid Diagnostics, Germany 2. Becton-Dickinson and Company, USA 3. Coris BioConcept, Belgium 4. SD Biosensor, Republic of Korea	30–40	Rapid	760	722	38
Baro et al. [147]	Spain	N	nsp	LFIA	1. PanBioTM COVID-19 Ag Rapid test 2. CLINITEST^®^ Rapid COVID-19 Antigen Test 3. SARS-CoV-2 Rapid Antigen Test 4. SARS-CoV-2 Antigen Rapid Test Kit 5. COVID-19 Coronavirus Rapid Antigen Test Cassette	1. Abbott 2. Siemens 3. Roche 4. Lepu Medica 5. Surescreen	Up to 30	Rapid	286	101	185
Caputo et al. [148]	Italy	N	nsp-ts	CLEIA	Lumipulse G SARS-CoV-2 Ag	Fujirebio, Tokio, Japan	Up to 40	Quick/detector	4266	503	3763
Kenyeres et al. [149]	Hungary	N	nsp	LFIA	BIOCREDIT COVID-19 Ag	RapiGEN Inc.	Up to 30	Rapid	37	37	NA
Häuser et al. [150]	Germany	N	nsp	CLEIA/virus culture data	LIAISON SARS-CoV-2 antigen test	Diasorin	20–30	Detector	196	196	27
Lefever et al. [151]	Belgium	N	nsp	LFIA/virus culture data	Liaison antigen test	Diasorin	20–30	Rapid	410	200	210
Zacharias et al. [152]	Austria	N	nsp	LFIA	SARS-CoV-2 RAT	Roche	30–40	Rapid	30	24	6
Oh et al. [153]	Korea	N	nsp	LFIA	Standard Q COVID-19 Ag	SD Biosensor, Inc. Gyeonggi-do, Korea	Up to 30	Rapid	118	26	92
Asai et al. [154]	Japan	N	nsp	CLEIA	LUMIPULSE SARS-CoV-2 antigen kit	Fujirebio, Japan	30–40	Detector	305	63	242
Kweon et al. [155]	Korea	N	nsp	LFIA	1. AFIAS COVID-19 Ag 2. ichromaTM COVID-19 Ag	1. Boditech Med., Chuncheon-si, Gang-won-do, Republic of Korea 2. Boditech Med.	Up to 30/Up to 40/30–40	Rapid	167	167	NA
Menchinelli et al. [156]	Italy	N	nsp	CLEIA/virus culture data	LUMIPULSE SARS-CoV-2 antigen kit	Fujirebio, Japan	Up to 20/Up to 30/Up to 40/0–20/20–30/30–40	Detector	594	194	400
Sood et al. [157]	USA	N	nsp	LFIA	BinaxNOW rapid antigen test	Abbott	20–30	Rapid	774	226	548
Epstude et al. [158]	Germany	N	nsp	LFIA	SARS-CoV-2 Rapid Antigen test	Roche^®^	Up to 40	Rapid	30	30	NA
Smith et al. [91]	USA	N	nsp	FIA/virus culture data	SARS Sofia FIA rapid antigen tests	Quidel	Up to 40	Rapid/detector	286	286	NA
Berger et al. [159]	Switzerland	N	nsp	LFIA/virus culture data	1. PanbioTM COVID-19 Ag Rapid Test device 2. Standard Q Ag-RDT	1. Abbott 2. SD Biosensor, Roche	20–30	Rapid	1064	315	749
Matsuda et al. [160]	Brazil	N	nsp	LFIA	1. COVID-19 Ag ECO Test 2. Panbio COVID-19 Ag Rapid Test	1. ECO Diagnóstica 2. Abbott, Ludwigshafen, Germany	Up to 40	Rapid	108	29	80
Van Honacker et al. [161]	Belgium	N	nsp	LFIA	1. COVID-19 ag BSS 2. SARS-CoV-2 Ag card 3. Coronavirus AG Rapid test cassette 4. Panbio COVID-19 Ag Rapid Test Device 5. SARS-CoV-2 Rapid Antigen test	1. Biosynex, Fribourg, Switzerland 2. Biotical health, Madrid, Spain 3. Zhejiang Orient Gene Biotech Co., Zhejiang, China 4. Abbott, Ludwigshafen, Germany 5. SD Biosensor, Gyeonggi-do, Korea	Up to 20/Up to 30/Up to 40/0–20/20–30/30–40	Rapid	98	58	40
Boum et al. [162]	Cameroon	N	nsp	LFIA	SARS-CoV-2 Rapid Antigen test	SD Biosensor	20–30	Rapid	1090	291	799
Mboumba Bouassa et al. [163]	France	N	nsp	LFIA	SIENNA™ COVID-19 Antigen Rapid Test Cassette	Salofa Oy, Salo, Finland; manufactured under license of T&D Diagnostics Canada Pvt. Ltd., Halifax, Canada	Up to 20/Up to 40	Rapid	150	100	50
Stokes et al. [164]	Canada	N	1. nsp 2. ts	LFIA	Panbio COVID-19 antigen Rapid Test Device	Abbott, IL, USA	Up to 40	Rapid	1888	497	1391
Landaas et al. [165]	Norway	N	nsp-ts	LFIA	Panbio™ COVID-19 Ag Rapid Test Device	Abbott	Up to 30/Up to 40/30–40	Rapid	3991	250	3741
Takeuchi et al. [166]	Japan	N	nsp	LFIA/virus culture data	QuickNavi™-COVID19 Ag	Denka Co., Ltd., Tokyo, Japan	Up to 40	Rapid	1186	105	1081
Igloi et al. [167]	Netherlands	N	nsp	LFIA/virus culture data	Roche SD Biosensor SARS-CoV-2 rapid antigen test	Roche Diagnostics	Up to 30/Up to 40/30–40	Rapid	970	186	784
Masiá et al. [168]	Spain	N	1. nsp 2. ts	LFIA	Panbio COVID-19 antigen Rapid Test Device	Abbott Rapid Diagnostic Jena GmbH, Jena, Germany	Up to 40	Rapid	2174	448	1726
Jääskeläinen et al. [169]	Finland	N	nsp	1. FIA 2. LFIA/virus culture data	1. Quidel Sofia SARS FIA 2. Standard Q COVID-19 Ag test 3. Panbio™	1. Quidel, San Diego, CA 2. SD Biosensor, Republic of Korea 3. Abbott Diagnostic GmbH, Jena, Germany	Up to 30/Up to 40/30–40	1. Rapid/detector 2. Rapid 3. Rapid	198	185	40
Olearo et al. [170]	Germany	N	nsp	LFIA/virus culture data	1. SARS-CoV-2 Rapid Antigen Test (Roche) 2. COVID-19 Rapid Test Device (Abbott) 3. MEDsan SARS-CoV-2 Antigen Rapid Test 4. CLINITEST Rapid COVID-19 Antigen Test	1. Roche Diagnostics SD Biosensor Korea 2. Abbott Rapid Diagnostics Panbio Ltd. Australia 3. MEDsan GmbH Germany 4. Zhejiang Orient Biotech Co. China	Up to 40	Rapid	184	84	100
Toshiaki Ishii et al. [171]	Japan	N	1. nsp 2. ts	1. LFIA 2. CLEIA	1. Espline^®^ SARS-CoV-2 2. Lumipulse^®^ SARS-CoV-2	1. Fujirebio Inc., Tokyo, Japan 2. Fujirebio Inc., Tokyo, Japan	Up to 20/Up to 30/Up to 40/0–20/20–30/30–40	1. Rapid 2. Quick/detector	893	44	849
Peña-Rodríguez et al. [172]	Mexico	N	nsp	LFIA	STANDARD™ Q COVID-19 Ag Test	SD BIOSENSOR	Up to 40	Rapid	369	104	265
Gili et al. [173]	Italy	N	nsp	CLEIA	Lumipulse^®^ SARS-CoV-2 antigen assay	Fujirebio, Inc., Tokyo, Japan	Up to 40	Quick/detector	1964	185	1779
Pérez-García et al. [174]	Spain	N	nsp	LFIA	1. CerTest SARS-CoV-2 Ag One Step Card Test 2. Panbio COVID-19 Ag Rapid Test Device	1. Certest Biotec S. L., Zaragoza, Spain 2. Abbot Rapid Diagnostics GmbH, Jena, Germany	Up to 30/Up to 40/30–40	Rapid	320	170	150
Kilic et al. [175]	USA	N	nsp	LFIA	BD Veritor SARS-CoV-2	Becton, Dickinson, Sparks, MD, USA	Up to 40	Rapid	1384	116	1268
Drain et al. [176]	USA	N	nsp	FIA	LumiraDx SARS-CoV-2 antigen test	LumiraDx UK Ltd., Dumyat Business Park, Alloa, FK10 2PB, UK)	Up to 40	Rapid/detector	512	123	389
Basso et al. [177]	Italy	N	1. nsp 2. ts	1. LFIA 2. LFIA 3. CLEIA	1. ESPLINE rapid test 2. COVID-19 Ag Rapid Test 3. Lumipulse G SARS-CoV-2 Ag	1. Fujirebio 2. ABBOTT 3. Fujirebio	Up to 40	1. Rapid 2. Rapid 3. Quick/detector	234	87	147
Pollock et al. [178]	USA	N	nsp	LFIA	BinaxNOW COVID-19 Ag card	Abbott Diagnostics Scarborough, Inc.	Up to 30/Up to 40/30–40	Rapid	2307	292	2015
Ristić et al. [179]	Serbia	N	nsp	LFIA	STANDARD Q COVID-19 Ag Test	SD Biosensor, Gyeonggi-do, Korea	Up to 40	Rapid	120	43	77
Courtellemont et al. [180]	France	N	nsp	LFIA	COVID-VIRO^®^	AAZ, Boulogne Billancourt, France	Up to 30/Up to 40/30–40	Rapid	248	121	127
Thommes et al. [181]	Austria	N	nsp	LFIA	1. Panbio™ COVID-19 Ag Rapid test 2. Novel Coronavirus (2019-nCov) Antigen Detection Kit 3. DIAQUICK COVID-19 Ag Cassette 4. SARS-CoV-2 Rapid Antigen Test	1. Abbott, Chicago, Illinois 2. CLMSRDL, Sichuan Mass Spectrometry Biotechnology Co., Ltd., Chengdu, Sichuan 3. DIALAB, Wiener Neudorf, Austria 4. Roche Diagnostics Deutschland GmbH, Mannheim, Germany	Up to 30/Up to 40/30–40	Rapid	154	154	NA
González-Donapetry et al. [182]	Spain	N	nsp	LFIA	Panbio COVID-19 Ag Rapid Test Device	Abbott Rapid Diagnostics Jena GmbH, Jena, Germany	Up to 40	Rapid	440	18	422
Eshghifar et al. [183]	?	N	ts	LFIA	1. BD Veritor™ System for rapid detection of SARS-CoV-2 2. CareStart™ COVID-19 Antigen 3. SG Diagnostics Antigen detection kit 4. Sofia SARS Antigen FIA 5. Rapid Response™ COVID-19 Antigen Rapid Test 6. Shenzhen SARS-CoV-2 Antigen Test kit 7. Genedia W COVID-19 Ag	1. Becton, Dickinson and Company, MD, USA 2. Accesas Bio, Inc., NJ, USA 3. SG Diagnostics, Singapore 4. Quedel Corporation, Hannover, Germany 5. BNTX, Inc., ON, Canada 6. Shenzhen Ultra-Diagnostics Biotec. Co., Ltd., Shenzhen, PRC 7. Green Cross Medical Sciences Corp., Chungcheongbuk, Republic of Korea	Up to 40	Rapid	5	5	NA
Merino et al. [184]	Spain	N	nsp	LFIA	Panbio™ COVID-19 Ag Rapid Test Device	Abbott Diagnostic GmbH, Jena, Germany	Up to 30/Up to 40/30–40	Rapid	958	359	599
Shao et al. [38]	USA	1. N 2. S	nsp	FET	In-house		Up to 40	NA/detector	38	28	10
Bulilete et al. [185]	Spain	N	nsp	LFIA	Panbio™ Ag-RDT	Abbott Diagnostic GmbH, Jena, Germany	Up to 40	Rapid	1367	140	1222
Torres et al. [186]	Spain	N	nsp	LFIA/virus culture data	CLINITEST® Rapid COVID-19 Antigen Test	Siemens, Healthineers, Erlangen, Germany	Up to 40	Rapid	270	116	154
Lindner et al. [187]	Germany	N	nsp	LFIA	STANDARD Q COVID-19 Ag Test	SD Biosensor, Inc., Gyeonggi-do, Korea	Up to 20/Up to 30/Up to 40/0–20/20–30/30–40	Rapid	179	41	138
Hirotsu et al. [188]	Japan	N	nsp	CLEIA	LUMIPULSE SARS-CoV-2 antigen test	Fujirebio, Inc., Tokyo, Japan)	Up to 40	Detector	1029	40	989
Salvagno et al. [189]	Italy	N	nsp-ts	LFIA	Roche SARS-CoV-2 Rapid Antigen Test	Roche Diagnostics, Basel, Switzerland	Up to 40	Rapid	321	149	172
Veyrenche et al. [190]	France	N	nsp	LFIA	Coris COVID-19 Ag Respi-Strip	BioConcept	Up to 30/Up to 40/30–40	Rapid	65	45	20
Porte et al. [191]	Chile	N	nsp	FIA	1. SOFIA SARS Antigen FIA 2. STANDARD F COVID-19 Ag FIA	1. Quidel Corporation, San Diego, CA, USA 2. SD Biosensor Inc., Gyeonggi-do, Republic of Korea	Up to 40	Rapid/detector	64	32	32
Domínguez Fernández et al. [192]	Spain	N	nsp	LFIA	Panbio™ rapid antigens test device	Abbott	Up to 40	Rapid	30	20	10
Kobayashi et al. [193]	Japan	N	nsp	1. CLEIA 2. LFIA	1. Lumipulse Presto SARS-CoV-2 Ag 2. Espline SARS-CoV-2	1. Fujirebio Inc., Tokyo, Japan 2. Fujirebio Inc., Tokyo, Japan	Up to 40	1. Quick/detector 2. Rapid	300	100	200
Houston et al. [194]	UK	N	nsp	LFIA	Innova SARS-CoV-2 Antigen Rapid Qualitative Test	Lotus Global Company, London, UK	Up to 40	Rapid	728	280	448
Gremmels et al. [73]	Netherlands/Aruba	N	nsp	LFIA	Panbio™ COVID-19 antigen	Abbott (Lake Country, IL, USA)	Up to 40	Rapid	1573	202	1371
Ciotti et al. [195]	Italy	N	nsp	LFIA	Coris COVID-19 Ag Respi-Strip	Coris BioConcept	Up to 40	Rapid	50	39	11
Okoye et al. [196]	USA	N	nsp	LFIA	Abbott BinaxNOW COVID-19 antigen card	Abbott Diagnostics Scarborough, Inc.	Up to 20/Up to 30/Up to 40/0–20/20–30/30–40	Rapid	2638	45	2593
Kurtulmus et al. [47]	Turkey	N	urine	UFT	In-house		Up to 40	Rapid	201	86	115
Saadi et al. [37]	France	N	nsp	1. LFIA 2. LFIA 3. LC-MS	1. NG Test Ag 2. COVID-19 Ag Respi-Strip 3. In-house	1. NG Biotech, France 2. Coris, Belgium	Up to 20/Up to 30/Up to 40/0–20/20–30/30–40	1. Rapid 2. Rapid 3. NA/detector	19	12	7
James et al. [197]	USA	N	nsp	LFIA	BinaxNOW COVID-19 Ag Card tests	Abbott Diagnostics, Scarborough	Up to 40	Rapid	2339	152	2187
Villaverde et al. [198]	Spain	N	nsp	LFIA	Panbio COVID-19 Ag Rapid Test	Abbott Rapid Diagnostic	Up to 40	Rapid	1620	77	1543
Pekosz et al. [199]	USA	N	nsp	LFIA/virus culture data	BD Veritor Antigen Test	Becton, Dickinson and Company, BD Life Sciences–, San Diego, California	Up to 40	Rapid	38	38	NA
Kohmer et al. [200]	Germany	N	nsp	LFIA/virus culture data	1. RIDA^®^QUICK SARS-CoV-2 Antigen 2. SARS-CoV-2 Rapid Antigen Test 3. NADAL^®^ COVID-19 Ag Test (test cassette) 4. LumiraDx™ Platform SARS-CoV-2 Ag Test	1. R-Biopharm AG, Darmstadt, Germany 2. Roche Diagnostics GmbH, Mannheim, Germany 3. Nal von Minden GmbH, Regensburg, Germany 4. LumiraDx GmbH, Cologne, Germany	Up to 40	Rapid	100	74	26
Prince-Guerra et al. [201]	USA	N	nsp	LFIA/virus culture data	BinaxNOW COVID-19 Ag Card	Abbott Diagnostics Scarborough, Inc.	Up to 40	Rapid	3419	299	3120
Möckel et al. [202]	Germany	N	nsp	LFIA/virus culture data	Roche SARS-CoV-2 rapid antigen test	SD Biosensor	Up to 40	Rapid	271	89	182
Rottenstreich et al. [203]	Israel	N	nsp	LFIA	NowCheck COVID-19 Ag Test	Bionote Inc., Hwaseong-si, Republic of Korea	Up to 30/Up to 40/30–40	Rapid	1326	9	1317
Favresse et al. [204]	Belgium	N	nsp	1. LFIA 2. LFIA 3. LFIA 4. LFIA 5. CLEIA	1. Biotical SARS-CoV-2 Ag card 2. Panbio™ COVID-19 Ag Rapid Test Device 3. Coronavirus Ag Rapid Test Cassette 4. Roche SARS-CoV-2 Rapid Antigen Test 5. VITROS Immunodiagnostic Products SARS-CoV-2 Antigen test	1. Biotical Health, Madrid, Spain 2. Abbott, Chicago, IL, USA 3. Healgen Scientific, Houston, TX, USA 4. Roche Diagnostics, Basel, Switzerland 5. Ortho Clinical Diagnostics, Raritan, NJ, USA	Up to 20/Up to 30/Up to 40/0–20/20–30/30–40	1. Rapid 2. Rapid 3. Rapid 4. Rapid 5. Quick/detector	188	96	92
Osterman et al. [205]	Germany	N	nsp-ts	1. LFIA 2. FIA	1. SARS-CoV-2 Rapid Antigen Test 2. STANDARD™ F COVID-19 Ag	1. SD Biosensor, Suwon, Korea 2. Roche, Switzerland	Up to 40	1. Rapid 2. Rapid/detector	1572	826	746
Pollock et al. [206]	USA	N	nsp	CLEIA/virus culture data	MSD S-PLEX SARS-CoV-2 N assay	MSD Meso Scale Discovery [MSD]	Up to 40	Quick/detector	226	136	90
Aoki et al. [207]	Japan	N	nsp	CLEIA	Lumipulse^®^ SARS-CoV-2 Ag	Fujirebio Inc., Tokyo, Japan	Up to 40	Quick/detector	548	30	518
Torres et al. [208]	Spain	N	nsp	LFIA	Panbio™ COVID-19 Ag	Abbott Diagnostics, Jena, Germany	Up to 40	Rapid	634	79	555
Alemany et al. [209]	Spain	N	nsp	LFIA	Panbio COVID-19 Ag Test	Abbott Rapid Diagnostics, Germany	Up to 30/Up to 40/30–40	Rapid	1406	951	455
Rastawicki et al. [210]	Poland	N	nsp	FIA	PCL COVID-19 Ag	PCL Inc., Korea	Up to 40	Rapid	42	36	6
Yamamoto et al. [211]	Japan	N	nsp	LFIA	ESPLINE SARS-CoV-2	Fujirebio Inc., Japan	Up to 40	Rapid	229	128	101
Kashiwagi et al. [212]	Japan	N	1. ts 2. nsp	LFIA	ESPLINE^®^ SARS-CoV-2	Fujirebio Inc., Tokyo	Up to 40	Rapid	6	4	2
Pilarowski et al. [213]	USA	N	nsp	LFIA/virus culture data	BinaxNOW rapid antigen test	Abbott Diagnostics Scarborough, Inc.	Up to 30/Up to 40/30–40	Rapid	871	26	845
Aoki et al. [214]	Japan	N	nsp	LFIA	Espline^®^ SARS-CoV-2	Fujirebio Inc., Japan	Up to 40	Rapid	129	63	66
Pray et al. [215]	Wisconsin	N	nsp	FIA/virus culture data	Sofia SARS Antigen	Quidel Corporation	Up to 40	Rapid/detector	1098	57	1041
Strömer et al. [216]	Germany	N	nsp	LFIA/virus culture data	1. NADAL^®^ COVID-19 Ag Test 2. Panbio™ COVID-19 Antigen	Nal von Minden GmbH, Moers, Germany Abbott Rapid Diagnostics, Germany	Up to 20/Up to 30/Up to 40/0–20/20–30/30–40	Rapid	124	124	NA
Toptan et al. [217]	Germany	N	nsp-ts	LFIA/virus culture data	novel antigen test	R-Biopharm	Up to 40	Rapid	67	58	9
Turcato et al. [218]	Italy	N	ts	LFIA	STANDARD Q COVID-19 Ag (R-Ag)	SD BIOSENSOR, KR	Up to 40	Rapid	3410	223	3187
Mak et al. [219]	Hong Kong	N	1. nsp-ts 2. nsp 3. ts	LFIA/virus culture data	Panbio COVID-19 Ag Rapid Test Device	Abbott Rapid Diagnostics, Germany	Up to 20/Up to 30/Up to 40/0–20/20–30/30–40	Rapid	35	8	27
Zhang et al. [220]	China	N	nsp-ts	FIA/virus culture data	SARS-CoV-2 N-protein test strip	Beijing Savant Biotechnology Co., Ltd.	Up to 40	Rapid/detector	547	247	300
Agulló et al. [221]	Spain	N	1. nsp 2. ts 3. nsp-ts	LFIA	Panbio COVID-19 Ag-RDT	Abbott Rapid Diagnostic Jena GmbH, Jena, Germany)	Up to 40	Rapid	659	126	527
Tanimoto et al. [222]	Japan	N	nsp	LFIA	ESPLINE SARS-CoV-2^®^	Fujirebio Inc., Tokyo, Japan	Up to 40	Rapid	8	2	6
Lindner et al. [223]	Germany	N	nsp	LFIA	STANDARD Q COVID-19 Ag Test	SD Biosensor, Inc., Gyeonggi-do, Korea	Up to 20/Up to 30/Up to 40/0–20/20–30/30–40	Rapid	39	39	NA
Abdelrazik et al. [224]	Egypt	N	nsp	LFIA	BIOCREDIT COVID-19 Ag test	RapiGEN Inc.	Up to 30/Up to 40/30–40	Rapid	188	188	NA
Weitzel et al. [225]	Chile	N	1. nsp-ts 2. nsp	1. LFIA 2. FIA 3. FIA	1. Biocredit One Step SARS-CoV-2 Antigen Test 2. Huaketai New Coronavirus (SARS-CoV-2) N Protein Detection Kit (FIA) 3. Diagnostic Kit for 2019-Novel Coronavirus (2019-nCoV)	1. RapiGen Inc., Anyang-si, Gyeonggi-do, Rep. of Korea 2. Savant Biotechnology Co., Beijing, China 3. Bioeasy Biotechnology Co., Shenzhen, China	Up to 40	1. Rapid 2. Rapid/detector 3. Rapid/detector	111	80	31
Winkel et al. [226]	Netherlands	N	nsp	LFIA	PanbioTM COVID-19 Ag	Abbott	Up to 40	Rapid	2390	63	2327
Hoehl et al. [227]	Germany	N	nsp	LFIA	RIDA^®^ QUICK SARS80 CoV-2 Antigen test	R-Biopharm	Up to 20	Rapid	602	8	594
Priya Kannian et al. [228]	India	N	nsp	LFIA	SARS-CoV2 antigen kit	SD Biosensor	Up to 40	Rapid	30	20	10
Lindner et al. [229]	Germany	N	nsp	LFIA	STANDARD Q COVID-19 Ag Test	SD Biosensor, Inc., Gyeonggi-do, Korea	Up to 40	Rapid	146	40	106
Filgueiras et al. [230]	Brazil	N	nsp	LFIA	SARS-CoV-2 rapid antigen test	ECODiagnostica	Up to 40	Rapid	139	55	84
Peto et al. [231]	UK	N	nsp-ts	LFIA	SARS-CoV-2 Antigen Rapid Qualitative Test	Innova	Up to 30	Rapid	834	198	636
Jakobsen et al. [232]	Denmark	N	nsp	LFIA	STANDARD Q COVID-19 Ag test	SD BIOSENSOR	Up to 40	Rapid	4811	221	4590
Miyakawa et al. [233]	Japan	N	nsp	LFIA/virus culture data	1. SARS-CoV-2 Ag-RDT 2. Panbio COVID-19 Ag Rapid Test 3. SARS-CoV-2 Rapid Antigen Test 4. SD Biosensor Standard Q COVID-19 Ag 5. Espline SARS-CoV-2	1. YCU-FF 2. Abbott 3. Roche 4. SD Bio 5. Fujirebio	Up to 40	Rapid	108	45	63
Torres et al. [186]	Spain	N	nsp	LFIA/virus culture data	CLINITEST^®^ Rapid 29 COVID-19 Antigen Test	Siemens, Healthineers, Erlangen, German	Up to 40	Rapid	270	33	237
Pollock et al. [234]	Massachusetts	N	nsp	LFIA	Access Bio CareStart COVID-19 Antigen test		Up to 30/Up to 40	Rapid	1498	234	1264
Shidlovskaya et al. [235]	Russia	N	nsp	LFIA/virus culture data	1. SGTI-flex COVID-19 Ag 2. Biocredit COVID-19 Ag	1. SUGENTECH, INC 2. RapiGEN Inc.	Up to 40	Rapid	106	14	92
Faíco-Filho et al. [236]	Brazil	N	nsp	LFIA	Panbio™ COVID-19 Ag Rapid Test	Abbott	Up to 30/Up to 40/30–40	Rapid	127	70	57
Schuit et al. [237]	Netherlands	N	nsp	LFIA/virus culture data	1. BD VeritorTM System Ag-RDT 2. SD Biosensor Ag-RDT	1. Becton, Dickinson and Company, Franklin Lakes, NJ, USA 2. Roche	Up to 40	Rapid	4274	365	4274
Ducrest et al. [238]	Switzerland	N	nsp	LFIA	COVIDia-Antigen	GaDia SA	Up to 30	Rapid	60	20	40
Vecchio et al. [239]	Italy	N	nsp	LFIA	Panbio™ COVID-19 Ag test	Abbott	Up to 30	Rapid	1441	61	1380
Bonde et al. [240]	Denmark	N	ts	LFIA	BD VERITOR Ag Rapid test	Becton-Dickinson and Company, USA	Up to 30	Rapid	809	65	744
Igloi et al. [241]	Netherlands	N	ts	LFIA/virus culture data	SARS-CoV-2 Rapid Antigen Test	Distributed by Roche (SD Biosensor)	Up to 30	Rapid	770	30	740
Thell et al. [242]	Austria	N	nsp	LFIA	SARS-CoV-2 Rapid Antigen Test	Roche Diagnostics	Up to 30	Rapid	541	213	328
Pollock et al. [243]	Massachusetts	N	nsp	LFIA	BinaxNOW COVID-19 Ag	Abbott	Up to 30	Rapid	98	98	NA
Hagbom et al. [244]	Sweden	N	ts	LFIA/virus culture data	1. Rapid Response™ COVID-19 Antigen Rapid Test Cassette for oral fluids 2. DIAGNOS™ COVID-19 Antigen Saliva Test	1. BioServ 2. DIAGNOS	Up to 30	Rapid	34	15	19
Thirion-Romero et al. [245]	Mexico	N	nsp	LFIA	Panbio™	Abbott	Up to 30	Rapid	1064	474	590
Chiu et al. [246]	Hong Kong	N	nsp	LFIA	INDICAID™ Rapid Test	PHASE Scientific i	Up to 30	Rapid	23,343	128	23,215
Abusrewil et al. [247]	Libya	N	nsp	LFIA	1. SARS-CoV-2 spike protein test 2. Shenzhen Microprofit Biotech Co 3. ESPLINE SARS-CoV-2 4. RapiGen COVID-19 Ag Detection Kit 5. Panbio™ COVID-19 Ag Rapid Test 6. Flowflex™ SARS-CoV-2 Antigen Rapid Test 7. Europe antigen testing COVID-19 8. Bioperfectus SARSCoV-2 Antigen Rapid Test Kit 9. AMP Rapid Test SARS-CoV-2 Ag 10. Coronavirus ag rapid test cassette	1. Fluorecare 2. Biotech 3. Fujirebio 4. Biocredit 5. Abbott 6. Acon 7. Assut 8. BIOPERFECTUS 9. AMP 10. Orient GENE	Up to 30/Up to 40	Rapid	231	83	145
Muthamia et al. [248]	Kenya	N	nsp	LFIA	BD Veritor antigen test	Becton-Dickinson and Company, USA	Up to 20/Up to 30/0–20/20–30	Rapid	272	47	225
Abdul-Mumin et al. [249]	Ghana	N	nsp	LFIA	STANDARD Q SARS-CoV-2 Ag Test	SD Biosensor	Up to 40	Rapid	193	42	151
Akashi et al. [250]	Japan	N	nsp	LFIA	QuickNavi™-COVID19 Ag	Otsuka Pharmaceutical Co., Ltd. (Otsuka) and Denka Company	Up to 40	Rapid	96	96	NA
Lindner et al. [251]	Germany	N	nsp	LFIA	1. Espline SARS-CoV-2 2. Sure Status COVID-19 Antigen Card Test 3. Mologic COVID-19 Rapid Test	1. Fujirebio Inc. 2. Premier Medical Corporation Private Limited 3. Fujirebio Inc	Up to 40	Rapid	329	329	NA
Suliman et al. [252]	Massachusetts	N	nsp	LFIA	Access Bio CareStart™ COVID-19 RDT	CareStart	Up to 30	Rapid	631	37	594
Bruins et al. [253]	Netherlands	N	nsp	LFIA	Panbio™ COVID-19 Ag Rapid Test	Abbott	Up to 30	Rapid	1101	84	917
Ford et al. [254]	Wisconsin	N	nsp	LFIA/virus culture data	BinaxNOW SARS-CoV-2 antigen test	Abbott Laboratories, Abbott Park, IL	Up to 40	Rapid	2110	334	1776
Koskinen et al. [255]	Finland	N	nsp	LFIA/virus culture data	mariPOC SARS-CoV-2 Antigen Test	mariPOC	Up to 30	Rapid/optional detector	211	13	198
Nikolai et al. [256]	Germany	N	nsp	LFIA	STANDARD Q COVID-19 Ag Test	SD Biosensor, Inc. Gyeonggi-do, Korea	Up to 40	Rapid	228	70	188
Stohr et al. [257]	Netherlands	N	nsp	LFIA/virus culture data	1. BD Veritor System for Rapid Detection of SARS-CoV-2 2. Roche SARS-CoV-2 antigen detection test	Becton Dickinson company, USA Roche, Switzerland	Up to 40	Rapid	3239	454	1528

LFIA: Lateral Flow Immunoassay; FIA: Fluorescence Immunoassay; CLEIA: Chemiluminescence Enzyme Immunoassay; FET: Field-Effect Transistors; Ag: Antigen; nsp: nasopharengeal; ts: oropharyngeal/throat/saliva; Rapid: detection time 5–20 min (mainly 15) but never exceeding 30 min; Quick: detection time 30–35 min; Quick *: 60 min; w/wo: with/without; Detector: a detector in needed to read the developed signal; NA: Not applicable; NR: Not reported; Cases: SARS-CoV-2 positive samples according to RT-PCR; Controls: healthy individuals and RT-PCR negative (for SARS-CoV-2); Virus culture data: study that provides any kind of data on the correlation between virus culture [cytopathic effect, tissue culture infective dose 50% (TCID 50), limit of detection (LoD)], and rapid Antigen Test positivity, RNA copies number, Ct values of RT-PCR positive samples.

**Table 2:** Results of the multivariate meta-analysis for the different types of assays using different samples and stratified according to different cut-off rt-PCR values. Listed information includes the pooled sensitivity and specificity along with the 95% confidence intervals (NSP: pharyngeal, nasopharyngeal, nasal specimens, TS: throat, saliva, N: nucleocapsid protein, S: spike protein, M: membrane E: envelope, NS: nucleocapsid and Spike proteins).

Sample	Ag	Method	Ct Values	Studies/Patients/ Controls	Sensitivity (95% CI)	Specificity (95% CI)	Studies w/o Controls
NSP	N	LFIA	0–20	41/7464/3945	0.945 (0.930, 0.961)	0.993 (0.987, 0.998)	22
NSP	N	LFIA	0–30	99/66,939/47,719	0.853 (0.826, 0.879)	0.991 (0.988, 0.995)	44
NSP	N	LFIA	0–40	207/88,008/69,415	0.702 (0.676, 0.727)	0.990 (0.987, 0.993)	30
NSP	N	LFIA	20–30	46/7817/4360	0.790 (0.739, 0.841)	0.987 (0.976, 0.998)	35
NSP	N	LFIA	30–40	71/5150/911	0.329 (0.265, 0.393)	0.959 (0.923, 0.995)	51
TS	N	LFIA	0–20	5/90/NA	0.805 (0.599, 1.000)	-	5
TS	N	LFIA	0–30	10/2136/1756	0.636 (0.477, 0.795)	0.994 (0.989, 0.998)	5
TS	N	LFIA	0–40	23/10,249/9232	0.354 (0.238, 0.470)	0.996 (0.993, 0.998)	12
TS	N	LFIA	20–30	6/160/NA	0.394 (0.086, 0.702)	-	6
TS	N	LFIA	30–40	4/44/NA	0.085 (0.000, 0.176)	-	4
NSP-TS	N	LFIA	0–20	7/4240/3859	0.999 (0.000, 1.000)	0.999 (0.000, 1.000)	6
NSP-TS	N	LFIA	0–30	12/9229/8133	0.867 (0.792, 0.942)	0.999 (0.997, 1.000)	10
NSP-TS	N	LFIA	0–40	30/23,970/21,699	0.696 (0.638, 0.754)	0.992 (0.987, 0.996)	4
NSP-TS	N	LFIA	20–30	10/1995/1504	0.575 (0.279, 0.870)	0.997 (0.987, 1.000)	7
NSP-TS	N	LFIA	30–40	10/217/NA	0.417 (0.242, 0.593)	-	9
NSP	N	FIA	0–20	3/97/NA	0.935 (0.880, 0.990)	-	3
NSP	N	FIA	0–30	10/2221/421	0.807 (0.726, 0.889)	0.992 (0.979, 1.000)	6
NSP	N	FIA	0–40	29/36,425/33,718	0.707 (0.631, 0.783)	0.984 (0.970, 0.997)	1
NSP	N	FIA	20–30	3/598/NA	0.729 (0.544, 0.915)	-	3
NSP	N	FIA	30–40	12/2283/665	0.435 (0.190, 0.680)	0.983 (0.971, 0.995)	9
TS	N	FIA	0–40	2/114/31	0.162 (0.083, 0.241)	0.984 (0.941, 1.000)	1
NSP-TS	N	FIA	0–30	4/195/77	0.944 (0.904, 0.985)	0.975 (0.944, 1.000)	1
NSP-TS	N	FIA	0–40	11/2779/2018	0.691 (0.520, 0.862)	0.971 (0.953, 0.989)	2
NSP-TS	N	FIA	30–40	3/72/32	0.792 (0.434, 1.000)	0.969 (0.926, 1.000)	1
NSP	N	CLEIA	0–20	3/789/152	0.955 (0.907, 1.000)	0.997 (0.000, 1.000)	2
NSP	N	CLEIA	0–30	3/1268/111	0.980 (0.960, 0.999)	0.995 (0.000, 1.000)	2
NSP	N	CLEIA	0–40	21/7626/5910	0.818 (0.774, 0.862)	0.978 (0.968, 0.988)	1
NSP	N	CLEIA	20–30	4/378/68	0.900 (0.672, 1.000)	0.986 (0.960, 1.000)	2
NSP	N	CLEIA	30–40	4/416/261	0.515 (0.220, 0.810)	0.978 (0.957, 0.999)	2
TS	N	CLEIA	0–20	1/136/NA	0.875 (0.550, 1.000)	-	1
TS	N	CLEIA	0–30	1/136/NA	0.928 (0.738, 1.000)	-	1
TS	N	CLEIA	0–40	3/376/179	0.709 (0.359, 1.000)	0.977 (0.950, 1.000)	1
TS	N	CLEIA	20–30	1/3/NA	0.875 (0.550, 1.000)	-	1
TS	N	CLEIA	30–40	1/3/NA	0.667 (0.000, 1.000)	-	1
NSP-TS	N	CLEIA	0–40	1/4266/3763	0.867 (0.837, 0.896)	0.973 (0.968, 0.978)	0
NSP-TS	N	CLEIA	20–30	1/978/817	0.795 (0.733, 0.857)	0.997 (0.000, 1.000)	0
NSP	N	other	0–20	2/45/7	0.973 (0.921, 1.000)	0.9375 (0.769, 1.000)	1
NSP	N	other	0–30	4/219/51	0.923 (0.807, 1.000)	0.963 (0.890, 1.000)	1
NSP	N	other	0–40	8/1228/388	0.768 (0.643, 0.894)	0.915 (0.821, 1.000)	0
NSP	N	other	20–30	2/110/NA	0.842 (0.422, 1.000)	-	2
NSP	N	other	30–40	4/73/NA	0.540 (0.147, 0.934)	-	4
NSP	S	LFIA	0–20	1/90/49	0.976 (0.928, 1.000)	0.857 (0.000, 1.000)	0
NSP	S	LFIA	0–30	2/407/234	0.783 (0.627, 0.938)	0.942 (0.833, 1.000)	0
NSP	S	LFIA	0–40	2/129/54	0.848 (0.768, 0.930)	0.862 (0.771, 0.954)	0
NSP	S	LFIA	20–30	1/80/49	0.677 (0.513, 0.842)	0.857 (0.000, 1.000)	0
NSP	S	other	0–40	4/286/207	0.872 (0.780, 0.963)	0.911 (0.761, 1.000)	0
TS	S	other	0–40	3/96/42	0.817 (0.635, 1.000)	0.931 (0.856, 1.000)	0
TS	N, S	other	0–40	1/433/397	0.986 (0.949, 1.000)	0.962 (0.943, 0.981)	0
NSP-TS	S + E + M	other	0–40	1/94/49	0.955 (0.895, 1.000)	0.959 (0.904, 1.000)	0
URINE	N, S	other, FIA	0–40	3/271/145	0.715 (0.310, 1.000)	0.869 (0.647, 1.000)	0

**Table 3:** Results of the multivariate meta-analysis performed cumulatively for methods and/or antigen tested, in <30 and <40 Ct values. Listed information includes the pooled sensitivity and specificity along with the 95% confidence intervals (NSP: pharyngeal, nasopharyngeal, nasal specimens, TS: throat, saliva, oropharyngeal, N: nucleocapsid protein, S: spike protein, M: membrane E: envelope, NS: nucleocapsid and Spike proteins).

Sample	Ag	Method (LFIA, FIA, CLEIA)	Ct Values	Studies	Sensitivity (95% CI)	Specificity (95% CI)	Studies w/o Controls
NSP	NS	LFIA or FIA or CLEIA	30	118	0.858 (0.835, 0.881)	0.991 (0.987, 0.995)	53
NSP	NS	LFIA or FIA or CLEIA	40	325	0.726 (0.706, 0.746)	0.989 (0.987, 0.992)	39
TS	NS	LFIA or FIA or CLEIA	30	10	0.637 (0.478, 0.795)	0.994 (0.989, 0.998)	5
TS	NS	LFIA or FIA or CLEIA	40	36	0.438 (0.332, 0.547)	0.993 (0.987, 0.999)	14
NSP	NS	LFIA or FIA	30	114	0.854 (0.830, 0.878)	0.991 (0.987, 0.995)	50
NSP	NS	LFIA or FIA	40	303	0.718 (0.697, 0.739)	0.989 (0.987, 0.992)	38
TS	NS	LFIA or FIA	30	10	0.637 (0.478, 0.795)	0.994 (0.989, 0.998)	5
TS	NS	LFIA or FIA	40	32	0.395 (0.285, 0.505)	0.995 (0.993, 0.997)	13
NSP	NS	LFIA	30	101	0.852 (0.825, 0.878)	0.991 (0.987, 0.995)	44
NSP	NS	LFIA	40	269	0.715 (0.692, 0.738)	0.990 (0.987, 0.992)	35
TS	NS	LFIA	30	10	0.637 (0.478, 0.795)	0.994 (0.989, 0.998)	5
TS	NS	LFIA	40	29	0.408 (0.292, 0.523)	0.995 (0.993, 0.997)	12
NSP	NS	FIA	30	13	0.868 (0.813, 0.924)	0.991 (0.981, 1.000)	6
NSP	NS	FIA	40	35	0.730 (0.674, 0.785)	0.986 (0.976, 0.995)	3
TS	NS	FIA	30	-	-	-	-
TS	NS	FIA	40	2	0.162 (0.083, 0.242)	0.984 (0.941, 1.000)	1
NSP	NS	CLEIA	30	4	0.977 (0.955, 0.998)	0.995 (0.000, 1.000)	3
NSP	NS	CLEIA	40	23	0.816 (0.761, 0.870)	0.979 (0.971, 0.988)	1
TS	NS	CLEIA	30	-	-	-	-
TS	NS	CLEIA	40	3	0.720 (0.380, 1.000)	0.957 (0.889, 1.000)	1

**Table 4:** Results of the meta-analysis for the different types of assays for symptomatic and asymptomatic patients. Listed information includes the pooled sensitivity and specificity along with the 95% confidence intervals. (NSP: pharyngeal, nasopharyngeal, nasal specimens, TS: throat, saliva, N: nucleocapsid protein, S: spike protein, NS: nucleocapsid and Spike proteins).

Sample	Ag	Method	Ct	Studies	Sensitivity (95% CI)	Specificity (95% CI)	Studies w/o Controls
***SYMPTOMATIC INDIVIDUALS***							
NSP	N	LFIA	20	1	0.976 (0.911, 1.000)	-	1
NSP	N	LFIA	30	21	0.823 (0.765, 0.882)	0.993 (0.989, 0.997)	7
NSP	N	LFIA	40	44	0.753 (0.713, 0.794)	0.992 (0.987, 0.997)	7
NSP	N	LFIA	20–30	2	0.881 (0.765, 0.996)	-	2
NSP	N	LFIA	30–40	13	0.469 (0.228, 0.709)	0.947 (0.880, 1.000)	4
NSP	N	FIA	30	2	0.694 (0.509, 0.878)	0.996 (0.993, 0.998)	0
NSP	N	FIA	40	4	0.605 (0.292, 0.918)	0.948 (0.827, 1.000)	1
NSP	N	FIA	30–40	1	0.921 (0.868, 0.973)	0.923 (0.000, 1.000)	0
TS	N	LFIA	30	2	0.669 (0.119, 1.000)	0.998 (0.994, 1.000)	0
TS	N	LFIA	40	4	0.426 (0.029, 0.823)	0.986 (0.977, 0.996)	0
TS	N	LFIA	30–40	1	0.025 (0.000, 1.000)	0.5 (0.000, 1.000)	0
TS	N	FIA	40	1	0.083 (0.000, 1.000)	-	1
NSP-TS	N	LFIA	20	2	0.957 (0.889, 1.000)	-	2
NSP-TS	N	LFIA	30	4	0.873 (0.788, 0.958)	0.998 (0.993, 1.000)	3
NSP-TS	N	LFIA	40	11	0.767 (0.695, 0.836)	0.996 (0.992, 0.999)	3
NSP-TS	N	LFIA	20–30	2	0.901 (0.795, 1.000)	-	2
NSP-TS	N	LFIA	30–40	4	0.260 (0.142, 0.378)	0.500 (0.000, 1.000)	3
***ASYMPTOMATIC INDIVIDUALS***							
NSP	N	LFIA	30	15	0.665 (0.558, 0.772)	0.992 (0.981, 1.000)	6
NSP	N	LFIA	40	35	0.561 (0.499, 0.622)	0.995 (0.992, 0.998)	5
NSP	N	LFIA	20–30	1	0.371 (0.270, 0.471)	-	1
NSP	N	LFIA	30–40	10	0.233 (0.061, 0.405)	0.947 (0.880, 1.000)	6
NSP	N	FIA	30	5	0.808 (0.714, 0.901)	0.997 (0.989, 1.000)	3
NSP	N	FIA	40	6	0.782 (0.614, 0.949)	0.949 (0.904, 0.995)	1
NSP	N	FIA	30–40	2	0.734 (0.253, 1.000)	0.882 (0.774, 0.991)	1
TS	N	LFIA	30	2	0.484 (0.000, 1.000)	0.995 (0.986, 1.000)	0
TS	N	LFIA	40	9	0.167 (0.034, 0.301)	0.990 (0.974, 1.000)	6
TS	N	LFIA	30–40	1	0.050 (0.000, 0.185)	0.5 (0.000, 1.000)	0
TS	N	FIA	40	1	0.166 (0.000, 1.000)	0.984 (0.941, 1.000)	0
NSP-TS	N	LFIA	30	1	0.300 (0.136, 0.464)	0.997 (0.000, 1.000)	0
NSP-TS	N	LFIA	40	5	0.481 (0.291, 0.671)	0.997 (0.995, 0.998)	1
NSP-TS	N	LFIA	30–40	1	0.050 (0.000, 0.185)	0.997 (0.000, 1.000)	0
NSP-TS	N	FIA	40	1	0.850 (0.772, 0.928)	0.984 (0.941, 1.000)	0

## Supplementary Materials

The following supporting information can be downloaded at: https://www.mdpi.com/article/10.3390/diagnostics12061388/s1, Table S1: The QUADAS tool; File S1: QUADAS-2 assessment results for included studies.
